# [^18^F]FMISO PET/CT imaging of hypoxia as a non-invasive biomarker of disease progression and therapy efficacy in a preclinical model of pulmonary fibrosis: comparison with the [^18^F]FDG PET/CT approach

**DOI:** 10.1007/s00259-021-05209-2

**Published:** 2021-02-13

**Authors:** Julie Tanguy, Françoise Goirand, Alexanne Bouchard, Jame Frenay, Mathieu Moreau, Céline Mothes, Alexandra Oudot, Alex Helbling, Mélanie Guillemin, Philippe Bonniaud, Alexandre Cochet, Bertrand Collin, Pierre-Simon Bellaye

**Affiliations:** 1 INSERM U1231, Equipe HSP-pathies, 7 Boulevard Jeanne d’Arc, Dijon, France; 2 Centre de Référence Constitutif des Maladies Pulmonaires Rares de l’Adultes de Dijon, réseau OrphaLung, Filère RespiFil. Centre Hospitalier Universitaire de Bourgogne, Dijon, France; 3 Centre George François Leclerc, Service de médecine nucléaire, Plateforme d’imagerie et de radiothérapie précliniques, 1 rue du professeur Marion, Dijon, France; 4 Institut de Chimie Moléculaire de l’Université́ de Bourgogne, UMR CNRS 6302, Université de Bourgogne Franche-Comté, 21000 Dijon, France; 5 Oncodesign, 21076 Dijon Cedex, France; 6 ImVIA, EA 7535, Université de Bourgogne, Dijon, France

**Keywords:** [^18^F]FMISO, Hypoxia, Lung fibrosis, PET/CT, [^18^F]FDG

## Abstract

**Purpose:**

Idiopathic pulmonary fibrosis (IPF) is a progressive disease with poor outcome and limited therapeutic options. Imaging of IPF is limited to high-resolution computed tomography (HRCT) which is often not sufficient for a definite diagnosis and has a limited impact on therapeutic decision and patient management. Hypoxia of the lung is a significant feature of IPF but its role on disease progression remains elusive. Thus, the aim of our study was to evaluate hypoxia imaging with [^18^F]FMISO as a predictive biomarker of disease progression and therapy efficacy in preclinical models of lung fibrosis in comparison with [^18^F]FDG.

**Methods:**

Eight-week-old C57/BL6 mice received an intratracheal administration of bleomycin (BLM) at day (D) 0 to initiate lung fibrosis. Mice received pirfenidone (300 mg/kg) or nintedanib (60 mg/kg) by daily gavage from D9 to D23. Mice underwent successive PET/CT imaging at several stages of the disease (baseline, D8/D9, D15/D16, D22/D23) with [^18^F]FDG and [^18^F]FMISO. Histological determination of the lung expression of HIF-1α and GLUT-1 was performed at D23.

**Results:**

We demonstrate that mean lung density on CT as well as [^18^F]FDG and [^18^F]FMISO uptakes are upregulated in established lung fibrosis (1.4-, 2.6- and 3.2-fold increase respectively). At early stages, lung areas with [^18^F]FMISO uptake are still appearing normal on CT scans and correspond to areas which will deteriorate towards fibrotic lesions at later timepoints. Nintedanib and pirfenidone dramatically and rapidly decreased mean lung density on CT as well as [^18^F]FDG and [^18^F]FMISO lung uptakes (pirfenidone: 1.2-, 2.9- and 2.6-fold decrease; nintedanib: 1.2-, 2.3- and 2.5-fold decrease respectively). Early [^18^F]FMISO lung uptake was correlated with aggressive disease progression and better nintedanib efficacy.

**Conclusion:**

[^18^F]FMISO PET imaging is a promising tool to early detect and monitor lung fibrosis progression and therapy efficacy.

**Supplementary Information:**

The online version contains supplementary material available at 10.1007/s00259-021-05209-2.

## Introduction

Idiopathic pulmonary fibrosis (IPF) is a progressive disease of the lung, inducing significant morbidity through worsening dyspnoea and overall lung function decline. The cause of IPF remains elusive and treatment options are limited with only two approved drugs, nintedanib and pirfenidone, which demonstrate efficacy to slow down fibrosis progression but are unable to reverse or even stop the disease [1]. The prognosis of IPF is poor, with a median survival time of less than 5 years.

Despite major improvements, the diagnosis of IPF remains complex. While it is possible to validate the diagnosis of IPF with typical high-resolution computed tomography (HRCT) in the presence of a usual interstitial pneumonia (UIP) pattern, most patients may require lung biopsy, which is associated with a high morbidity and mortality [2]. In addition, honeycombing, one of the key imaging criteria of UIP, is indicative of an already advanced stage of the disease. Yet, early diagnosis is crucial in IPF since longer time to diagnosis is related to poorer outcome and increased mortality [3]. Thus, imaging biomarkers for early/active fibrosis are needed to promote early diagnosis, which could dramatically shorten current treatment delays.

The lack of tools to monitor disease progression and response to anti-fibrotic therapies are also major clinical concerns. IPF progression is heterogeneous with some patients deteriorating rapidly leading to death within months, while others follow a slower decline with limited progression. In addition, the high interpersonal variability among IPF patients in the response to pirfenidone and nintedanib suggests that non-responders to one drug might benefit from the other. Considering the disabling side effects which may be associated with these therapies, the early prediction of treatment efficacy is a major clinical issue. As a consequence, identifying novel imaging predictive biomarkers to predict/monitor fibrosis progression and therapeutic efficacy would be the first step towards a personalized management of IPF patients.

In vivo molecular imaging has become an essential tool in preclinical research, clinical trials and medical practice. Recent publications highlight potential benefits from the use of [^18^F]FDG PET imaging in IPF mainly due to the TGFβ1-dependent upregulation of glucose transporters on myofibroblasts [4]. Lung areas with high [^18^F]FDG uptake have been associated with regions of lung parenchymal changes on HRCT [5–7]. In addition, [^18^F]FDG lung uptake has been shown to be increased in a preclinical model of lung fibrosis induced by bleomycin (BLM) [8]. Interestingly, in BLM-treated mice, pirfenidone significantly reduced the [18F]-FDG uptake compared to control mice [8]. Despite such promising findings, whether [^18^F]FDG PET has a significant clinical value for the management of IPF patients remains unknown.

Tissue remodelling occurring during the course of IPF ultimately causes the disruption of the alveolar structure leading to major hypoxic areas in the lungs of IPF patients [9]. Several studies have demonstrated a direct link between hypoxia and the development of IPF, mainly because hypoxia worsens fibrosis progression by increasing ECM deposition and vice versa. Transcriptomic analysis of IPF lungs has shown that hypoxia signalling pathways are upregulated [10]. At the cellular level, hypoxia signalling is mainly mediated by the upregulation of HIF-1α which is able to drive the behaviour of IPF-derived fibroblasts. As a consequence, the scarring of lung tissue in IPF is reinforced by the expansion of fibroblast/myofibroblasts *foci*. Interestingly, HIF-1α and carbonic anhydrase IX (CAIX), a transmembrane protein expressed in hypoxic conditions, have been demonstrated to be upregulated in the lungs of IPF patients [11]. HIF-1α and CAIX expression have also been shown to be upregulated in preclinical models of lung fibrosis [12, 13], and chronic hypoxia was shown to worsen BLM-induced lung fibrosis in mice [14]. Furthermore, Burman et al. [15] demonstrated that BLM induced the formation of hypoxic patches localized around fibrotic areas. Pimonidazole, a compound which specifically accumulates in cells in hypoxic conditions, was found to accumulate in alveolar epithelial cells boarding fibrotic lung areas upon BLM in mice. Several hypoxia-sensing probes have been extensively studied in cancer and are now evaluated in preclinical and clinical settings as tracers for hypoxia imaging including ^18^F-fluoromisonidazole ([^18^F]FMISO [16]). Whether tracers of tissue hypoxia may be useful for lung fibrosis monitoring remains unstudied and preclinical data evaluating [^18^F]FMISO PET in lung fibrosis are desperately missing. We therefore hypothesized that hypoxia might be an early event in the course of lung fibrosis development and might drive fibrosis progression. The current study evaluates the value of hypoxia in vivo PET imaging with [^18^F]FMISO to monitor disease progression and therapy efficacy in a preclinical model of pulmonary fibrosis in comparison with computed tomography (CT) and [^18^F]FDG PET imaging.

## Material and methods

### Animal experiments

All animal studies were conducted in accordance with the legislation on the use of laboratory animals (directive 2010/63/EU) and were approved by an accredited ethical committee (C2ea Grand Campus n°105) and the French Ministries of Research and Agriculture (project #12816). Eight-week-old female C57/Bl6 mice received at D0 a single intratracheal injection of 2 mg/kg of BLM (Santa Cruz Biotechnology, USA) or NaCl (controls) under anaesthesia (3% isoflurane). BLM-induced lung fibrosis is characterized by an initial inflammatory phase lasting from D0 to D7 which moves on to a fibrotic phase from D7 to D23. When indicated, animals were treated with nintedanib (60 mg/kg) or pirfenidone (300 mg/kg) by daily gavage during the fibrotic phase of BLM-induced lung fibrosis from D9 to D23 as recommended [17].

### PET/CT imaging

A pilot study was performed on NaCl- (*n* = 3) and BLM-receiving (*n* = 6) mice by successive PET/CT with [^18^F]FDG at D20 and [^18^F]FMISO at D21. Mice were anaesthetized through isoflurane (1.5%) inhalation for intravenous injection (tail vein) of 5 MBq of [^18^F]FDG 20 min before imaging or 10 MBq of [^18^F]FMISO 2 h before imaging. Mice were then maintained under anaesthesia (1.5%) and placed on an imaging heated bed inside a BioPET™-CT (Bioscan, USA). A CT scan of a lung-centered region was obtained (150 μA, 45 kV, 360 projections, 2 shots/projection) followed by PET acquisition (30 min) of the same region (lung-centered, 250–700 keV, Suppl. Fig. 1a).

In another experiment, longitudinal imaging of lung fibrosis was performed on NaCl- (*n* = 4) and BLM-receiving mice treated with vehicle (*n* = 5), nintedanib (*n* = 5) or pirfenidone (*n* = 5) by successive PET/CT with [^18^F]FDG and [^18^F]FMISO before BLM installation, at D8/D9, D15/D16 and D22/D23 following the same imaging protocol as described above (Suppl. Fig. 1b).

After the last [^18^F]FMISO PET imaging, mice were sacrificed and lungs and blood were harvested for ex vivo quantification using a γ-counter (Wizard, Perkin Elmer). Lungs were collected in 10% formalin for further histological analysis.

### Image analysis

All PET/CT fusion images were obtained using the VivoQuant™ software (Invicro, USA). Each PET/CT image was visually interpreted and 3D regions of interest (3DROI) corresponding to the lungs were manually drawn for CT quantification and to determine their radioactivity content. Injected doses per animal were measured at the time of injection in MBq. Lung radioactivity content was expressed in MBq, converted to percentage of injected dose per gram of tissue (%ID/g). PET scanner was indeed cross-calibrated to the dose calibrator and PET data were corrected for scatter and attenuation. All images were decay corrected for quantification. Additionally, other PET parameters were measured for [^18^F]FDG and [^18^F]FMISO (SUVmean, SUVmax), lung-to-background ratio (LTBR). SUV values have been calculated using a general soft tissue value of 1 g/mL. In addition, the metabolic lung volume (MLV) which refers to the volume of the lungs with high metabolic activity was determined on [^18^F]FDG PET. The MLV represents the volume of lung tissue with a [^18^F]FDG uptake above a threshold determined at D0 as follows [18]:

$$ \mathrm{MLVthreshold}=\left(\mathrm{SUVmean}\right)\mathrm{D}0+2\ \mathrm{SD} $$

Finally, the hypoxic lung volume (HLV) was calculated exclusively on [^18^F]FMISO PET. The HLV represents the volume of lung tissue with a [^18^F]FMISO uptake above a threshold determined as follows [8]:

$$ \mathrm{HLVthreshold}=\mathrm{LTBR}\times 1.4 $$

In addition, a semi-automatic segmentation of 3DROI was performed on CT scans as follows: normal lung density (− 800 to − 100 HU) corresponding to aerated lung areas and high lung density (− 100 to 300 HU) corresponding to non-aerated/fibrotic lung areas as previously described [19]. This semi-automatic segmentation allowed the independent quantification of the radioactivity content of [^18^F]FDG and [^18^F]FMISO (%ID/g) in normal and high-density lung tissue respectively.

### Fluorescence in vivo imaging

Hypoxia fluorescence imaging was performed on NaCl- and BLM-receiving mice at D21. Mice under anaesthesia (1.5% isoflurane) received intravenous injection (tail vein) of a carboxic anhydrase IX (CAIX) targeting tracer, Hypoxisense 680™ (2 nmol/100 μL), 24 h prior imaging. Hypoxisense 680™ (PerkinElmer, France) is a commercially available near-infrared (NIR) fluorescent agent which detects the surface expression of CAIX) protein, which increases in hypoxic tissues. Mice were then maintained under anaesthesia (1.5% isoflurane) and placed on an imaging heated bed inside an optical imager IVIS® LUMINA III (Perkin Elmer, France). Fluorescence images were acquired with the optimal filter pair (*λ*_ex_: 660 nm, *λ*_em_: 710 nm). Images were processed through the Living Image® software (Perkin Elmer) for exclusion of background signals (auto-fluorescence, food). Data were quantified with the Living Image® software and expressed in mean radiant efficiency.

### Collagen quantification

For histomorphometric assay, the amount of collagen in paraffin-embedded tissue sections was quantified by staining with Picrosirius red as previously described [20].

### Immunohistochemistry-immunofluorescence

After deparaffinization (Xylene), endogenous peroxidases inhibition (PBS-H_2_O_2_ 1% 20 min) and antigen unmasking (30 min in citrate buffer pH 6), sections were saturated (BSA 8%) and incubated overnight at 4 °C with specific antibodies at a dilution of 1:100 for the detection of HIF-1α (Santa Cruz Biotechnology, sc-13515), GLUT-1 (Abcam, ab115730) and α-SMA (Abcam, ab21027). HRP-conjugated antibody (Jackson ImmunoReasearch Laboratories, Suffolk, UK) or Alexa488- and Alexa568-conjugated (Abcam, France) were used as the secondary antibody (1:250, 45 min). For immunofluorescence, slides were mounted in ProLong® Gold with DAPI (ProLong® Gold antifade reagent with DAPI, Life Technologies).

### Statistical analysis

Comparison between two groups was performed using the Mann-Whitney non-parametric *t* tests. Comparison between multiple groups has been performed using the Kruskal-Wallis non-parametric ANOVA tests. A *p* < 0.05 was considered significant (*p < 0.05, **p < 0.01, ***p < 0.001). Results are presented as mean ± SEM.

## Results

### CT, [^18^F]FDG and [^18^F]FMISO PET are able to detect BLM-induced lung fibrosis

**Fig. 1:**
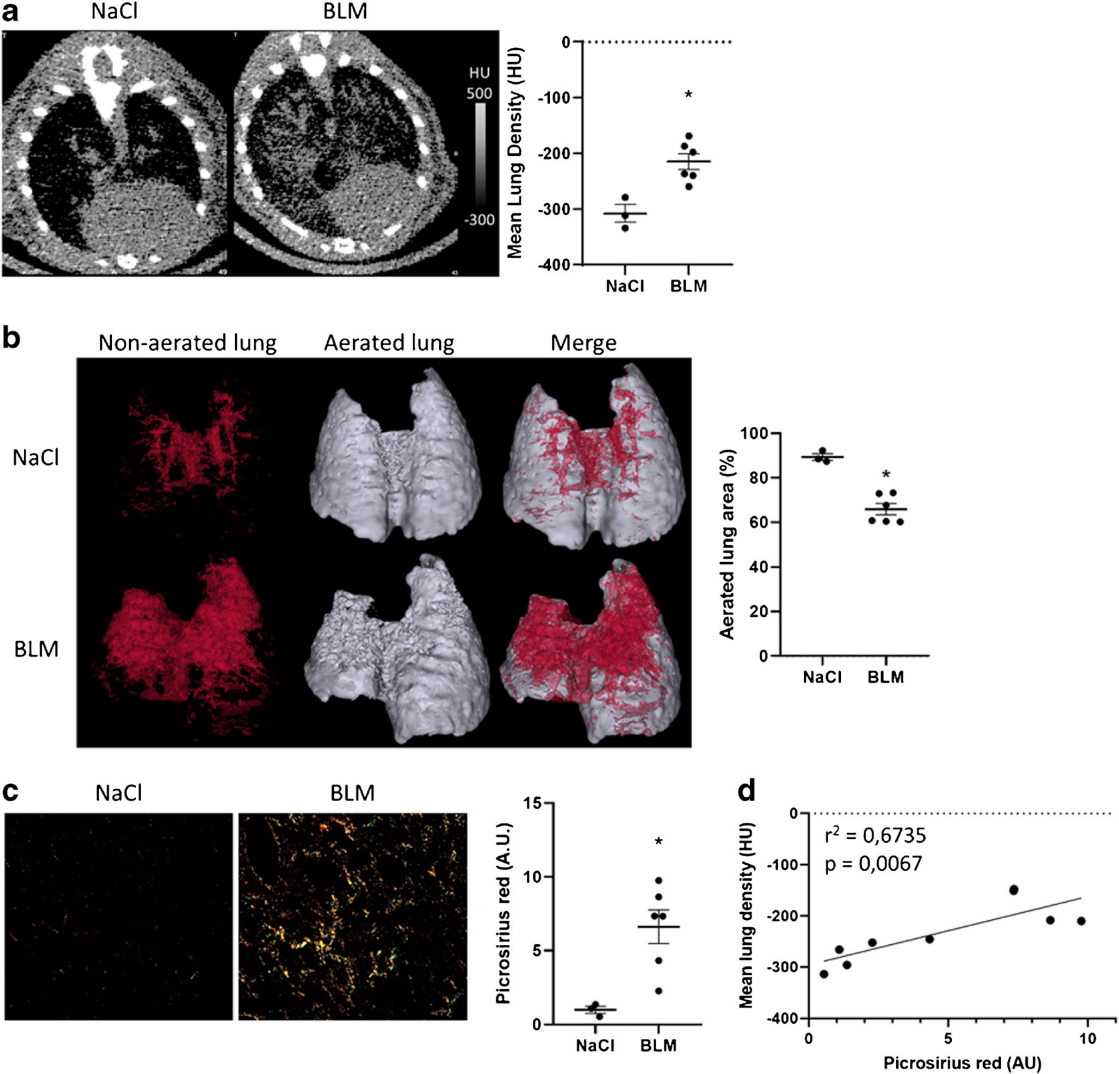
Computed tomography is able to detect advanced BLM-induced lung fibrosis. **a** Representative lung CT images of NaCl- and BLM-receiving mice at D21. Graph represents the mean lung density quantified on CT images. Results are presented as mean ± SEM, *n* = 3 for NaCl and *n* = 6 for BLM. *p < 0.05. HU, Hounsfield unit. **b** Representative 3D reconstruction of lung 3DROI segmentation of lung CT images of NaCl- and BLM-receiving mice at D21. Red represents high-density lung areas (− 100 to 300 HU) representative of non-aerated lungs; grey represents normal density lung areas (− 800 to − 100 HU) representative of aerated lungs. Graph represents the percentage of aerated lung on CT images. Results are presented as mean ± SEM, *n* = 3 for NaCl and *n* = 6 for BLM, *p < 0.05. **c** Representative Picrosirius red staining lung sections of NaCl- and BLM-receiving mice at D21. Graph represents the intensity of picrosirius red staining. Results are presented as mean ± SEM, *n* = 3 for NaCl and *n* = 6 for BLM, *p < 0.05. **d** Correlation between mean lung density (HU) measured on CT images and picrosirius red staining of corresponding lungs at D21

BLM-receiving mice (or NaCl) underwent successive [^18^F]FDG PET/CT and [^18^F]FMISO PET/CT at D20 and D21, respectively. Lung CT of BLM-receiving mice at D21 showed an increase in fibrotic consolidations compared with control mice (Fig. 1a). CT quantification showed an increase in mean lung density (MLD) in BLM-treated mice compared with controls (Fig. 1a). In addition, lungs from BLM-treated mice presented an increase in high-density voxels associated with a decrease in normal density voxels showing a limitation of aerated lung areas upon BLM (Fig. 1b). As expected, BLM induced an increase in lung collagen content measured by Picrosirius red staining (Fig. 1c). Interestingly, MLD measured on CT images strongly correlated with lung collagen content (Fig. 1d).

**Fig. 2:**
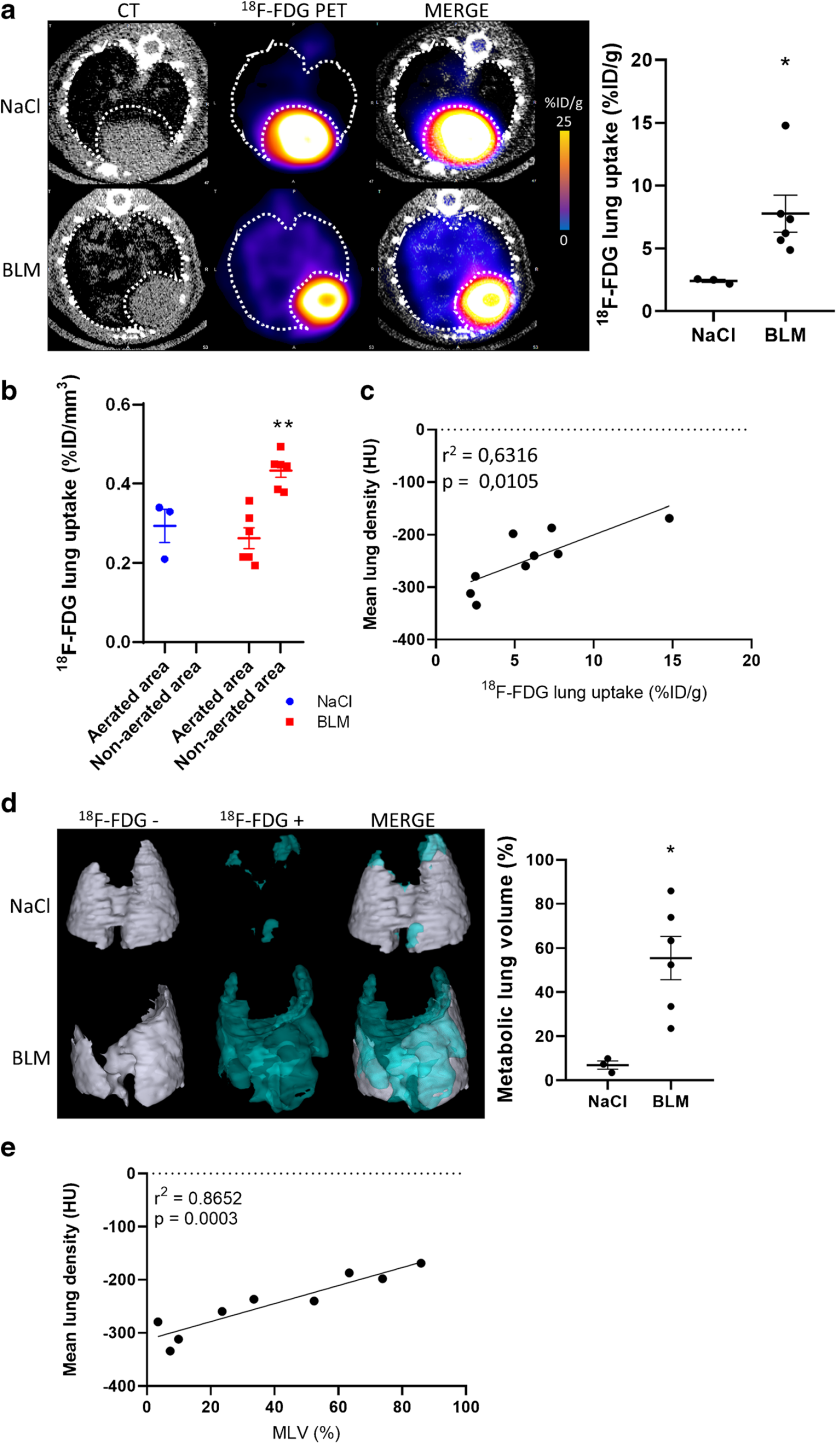
[^18^F]FDG is able to detect advanced BLM-induced lung fibrosis. **a** Representative lung PET/CT images with [^18^F]FDG of NaCl- and BLM-receiving mice at D21. Graph represents the [^18^F]FDG lung uptake in %ID/g of NaCl- and BLM-receiving mice at D21. Results are presented as mean ± SEM, *n* = 3 for NaCl and *n* = 6 for BLM. *p < 0.05. **b** Graph represents the [^18^F]FDG lung uptake in %ID/g of NaCl- and BLM-receiving mice at D21 in aerated and non-aerated lung areas (segmented on CT images). Results are presented as mean ± SEM, *n* = 3 for NaCl and *n* = 6 for BLM. **p < 0.01. **c** Correlation between mean lung density (HU) measured on CT images and [^18^F]FDG lung uptake (%ID/g) of corresponding lungs measured on PET at D21. **d** Representative 3D reconstruction of lung 3DROI segmentation of lung PET images representative of metabolic lung volume (MLV) of NaCl- and BLM-receiving mice at D21. Cyan represents areas with high [^18^F]FDG lung uptake (above the threshold of MLV_threshold_ = (SUVmean)_D0_ + 2SD); grey represents areas with low [^18^F]FDG lung uptake (below the threshold). Graph represents the MLV of NaCl- and BLM-receiving mice at D21. Results are presented as mean ± SEM, *n* = 3 for NaCl and *n* = 6 for BLM. *p < 0.05. **e** Correlation between mean lung density (HU) measured on CT images and MLV (%) of corresponding lungs measured on PET at D21

**Fig. 3:**
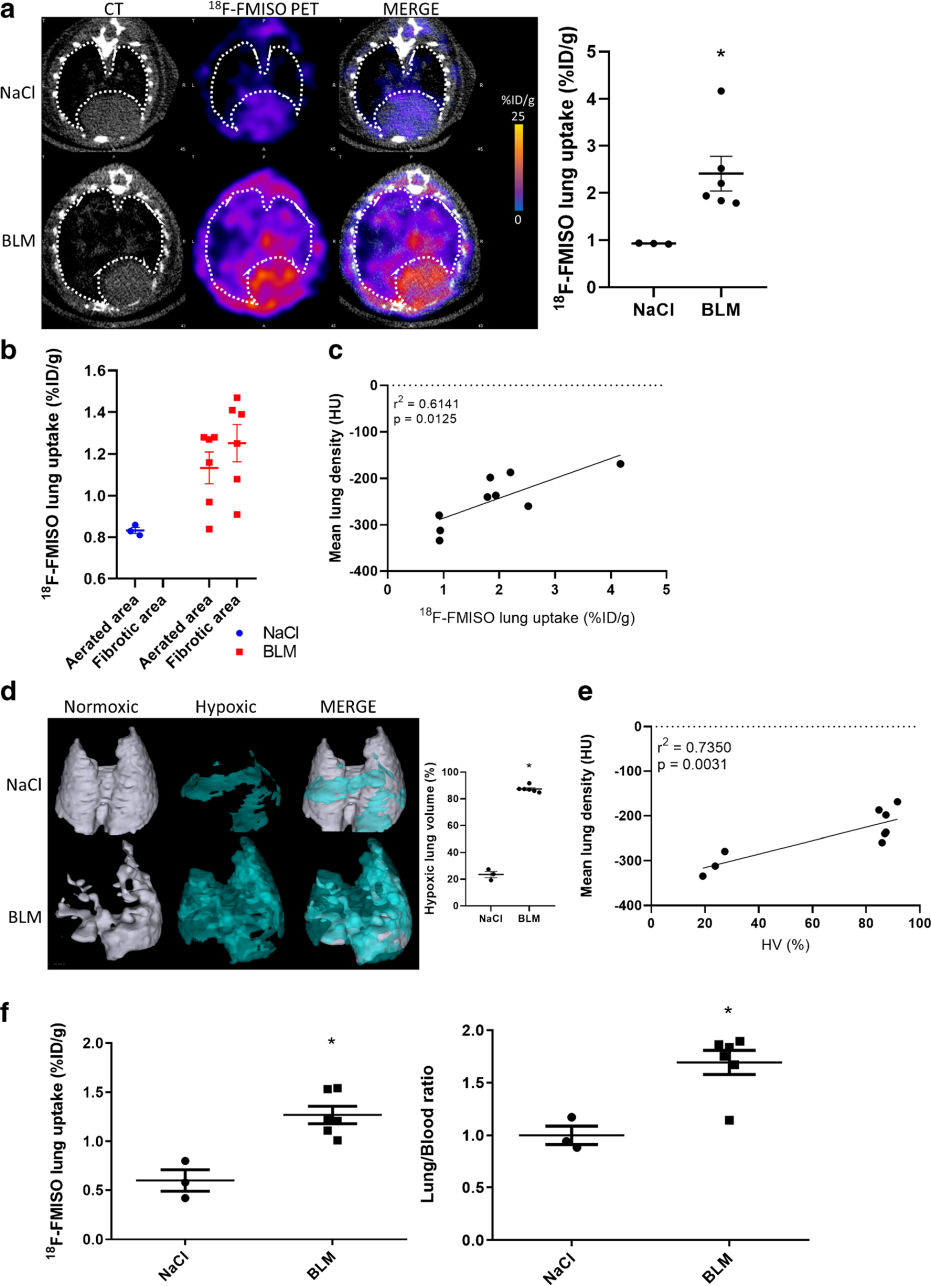
[^18^F]FMISO is able to detect advanced BLM-induced lung fibrosis. **a** Representative lung PET/CT images with [^18^F]FMISO of NaCl- and BLM-receiving mice at D21. Graph represents the [^18^F]FMISO lung uptake in %ID/g of NaCl- and BLM-receiving mice at D21. Results are presented as mean ± SEM, *n* = 3 for NaCl and *n* = 6 for BLM. *p < 0.05. **b** Graph represents the [^18^F]FMISO lung uptake in %ID/g of NaCl- and BLM-receiving mice at D21 in aerated and non-aerated lung areas (segmented on CT images). Results are presented as mean ± SEM, *n* = 3 for NaCl and *n* = 6 for BLM. **c** Correlation between mean lung density (HU) measured on CT images and [^18^F]FMISO lung uptake (%ID/g) of corresponding lungs measured on PET at D21. **d** Representative 3D reconstruction of lung 3DROI segmentation of lung PET images representative of hypoxic lung volume (HLV) of NaCl- and BLM-receiving mice at D21. Cyan represents areas with high [^18^F]FMISO lung uptake (above the threshold of HLV_threshold_ = TBR × 1.4); grey represents areas with low [^18^F]FMISO lung uptake (below the threshold). Graph represents the MLV of NaCl- and BLM-receiving mice at D21. Results are presented as mean ± SEM, *n* = 3 for NaCl and *n* = 6 for BLM. *p < 0.05. **e** Correlation between mean lung density (HU) measured on CT images and HLV (%) of corresponding lungs measured on PET at D21. **f** Radioactivity content in lungs of NaCl- and BLM-receiving mice and lung to blood ratio at D21 measured ex vivo with a γ-counter. Results are presented as mean ± SEM, *n* = 3 for NaCl and *n* = 6 for BLM. *p < 0.05

The global [^18^F]FDG and [^18^F]FMISO lung uptake was significantly increased in BLM-treated mice at D20 and D21 respectively compared to controls (Fig. 2a, Fig. 3a). Similarly, SUVmean, SUVmax and LTBR were increased in BLM-treated mice at D20 and D21 (Suppl Fig. 2a, Suppl Fig. 3a). Interestingly, the increase in [^18^F]FDG uptake was mainly localized in high-density lung areas observed on CT images confirming a specific uptake of [^18^F]FDG in fibrotic lung areas (Fig. 2b). Contrarily, the increase in [^18^F]FMISO uptake was localized in both aerated and non-aerated lung areas suggesting an uptake of [^18^F]FMISO in lung areas appearing normal on CT scans (Fig. 3b). In addition, [^18^F]FDG and [^18^F]FMISO lung uptake was strongly correlated with MLD measured on CT (Fig. 2c, Suppl Fig. 2b, Fig. 3c, Suppl Fig. 3b). Moreover, upon BLM, the MLV and HLV significantly increased (Fig. 2d, Fig. 3d) with a very strong correlation with MLD quantified on CT (Fig. 2e).

Finally, ex vivo radioactivity quantification of the lungs confirmed an increase in [^18^F]FMISO lung uptake at D21 in BLM-treated animals associated with an increase in lung/blood ratio (Fig. 3f).

The hypoxic status of the lungs of BLM-treated animals was confirmed by optical imaging using Hypoxisense™. The lung uptake of Hypoxisense™ was increased at D21 demonstrating that CAIX expression was enhanced in fibrotic lungs (Suppl Fig. 4a–b). Interestingly, the Hypoxisense™ signal was correlated with lung collagen content (Suppl Fig. 4c).

#### CT can assess anti-fibrotic therapies efficacy

**Fig. 4:**
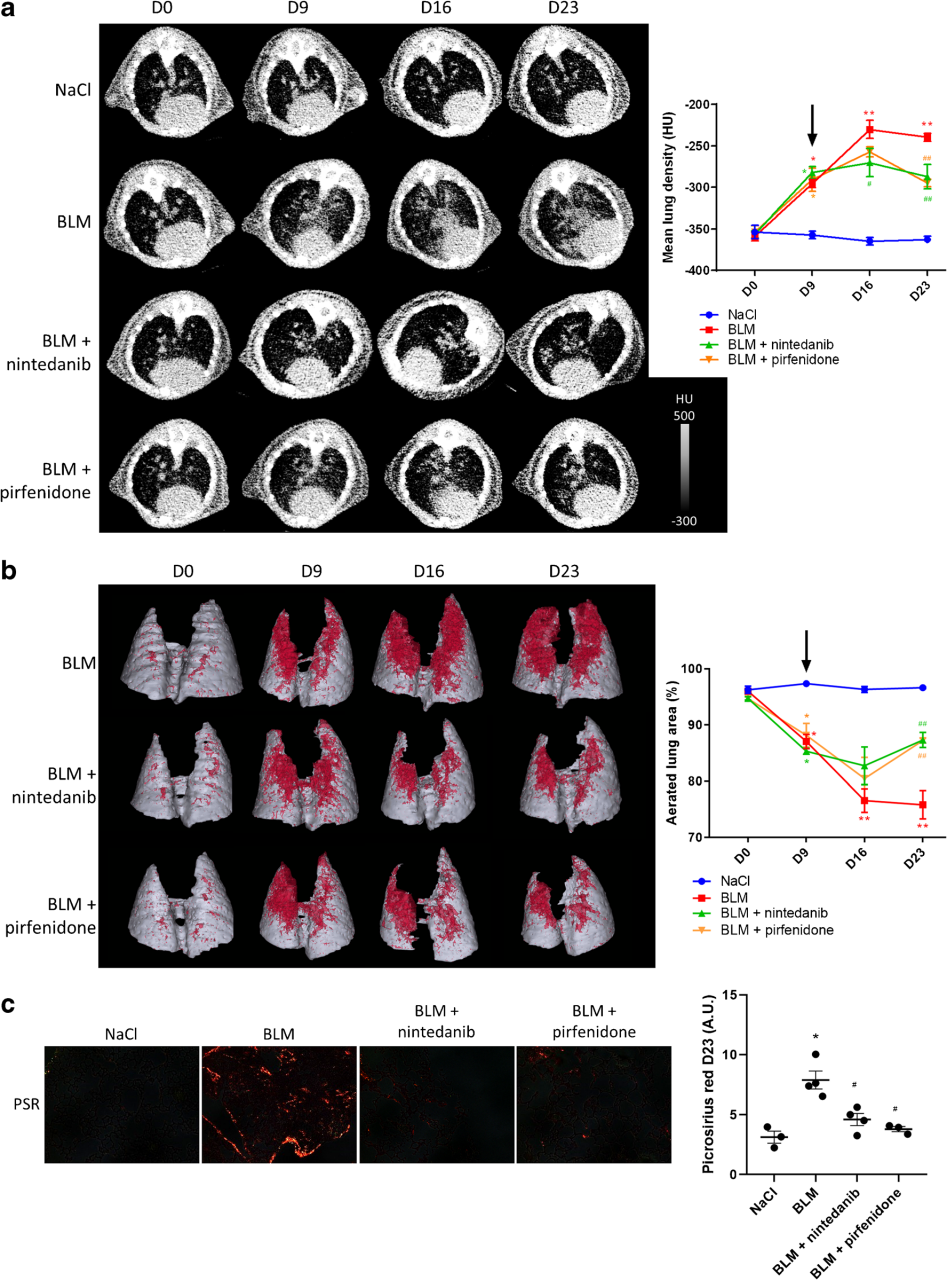
CT scan can detect lung fibrosis and efficacy of anti-fibrotic therapies. **a** Representative lung CT images of NaCl- and BLM-receiving mice treated or not with nintedanib of pirfenidone at D0, D9, D16 and D23. Graph represents evolution of the mean lung density quantified on CT images at all time points. Results are presented as mean ± SEM, *n* = 4 for NaCl and *n* = 5 for other groups. Stars (*) are representative of statistical comparison between time points for each group and hashes (#) are representative of statistical comparison between the groups at each time points. *^(#)^*p* < 0.05, **^(##)^*p* < 0.01. Black arrow represents the start of treatments. **b** Representative 3D reconstruction of lung 3DROI segmentation of lung CT images of NaCl- and BLM-receiving mice treated or not with nintedanib of pirfenidone at D0, D9, D16 and D23. Red represents high density lung areas (− 100 to 300 HU) representative of non-aerated lungs; grey represents normal density lung areas (− 800 to − 100 HU) representative of aerated lungs. Graph represents the percentage of evolution of aerated lung on CT images. Results are presented as mean ± SEM, *n* = 4 for NaCl and *n* = 5 for other groups. Stars (*) are representative of statistical comparison between time points for each group and hashes (#) are representative of statistical comparison between the groups at each time points. *p < 0.05, **^(##)^*p* < 0.01. Black arrow represents the start of treatments. **c** Representative Picrosirius red staining lung sections of NaCl- and BLM-receiving mice treated or not with nintedanib or pirfenidone at D23. Graph represents the intensity of picrosirius red staining. Results are presented as mean ± SEM, *n* = 3 for NaCl, *n* = 4 for BLM and BLM-nintedanib and *n* = 3 for BLM-pirfenidone. Stars (*) are representative of the comparison of each group with the NaCl group and hashes (#) are representative of statistical comparison of each group with BLM group *^(#)^*p* < 0.05

CT scan revealed an increase in MLD in BLM-treated mice starting from D9 with a peak at D16 and a stabilization up to D23 (Fig. 4a). CT quantification showed that pirfenidone and nintedanib stopped the increase in MLD induced by BLM from D9 to D23 (Fig. 4a). A deeper analysis demonstrated that BLM induces fibrosis progression with a decrease in lung aerated areas which was prevented by pirfenidone and nintedanib from D9 to D23 (Fig. 4b, Suppl Fig. 5). CT results at D23 were confirmed by histology which showed an increase in collagen in BLM-treated mice (Fig. 4c) which was inhibited by nintedanib and pirfenidone (Fig. 4c).

#### [^18^F]FDG PET can detect advanced lung fibrosis and is suitable for anti-fibrotic therapies follow-up

**Fig. 5:**
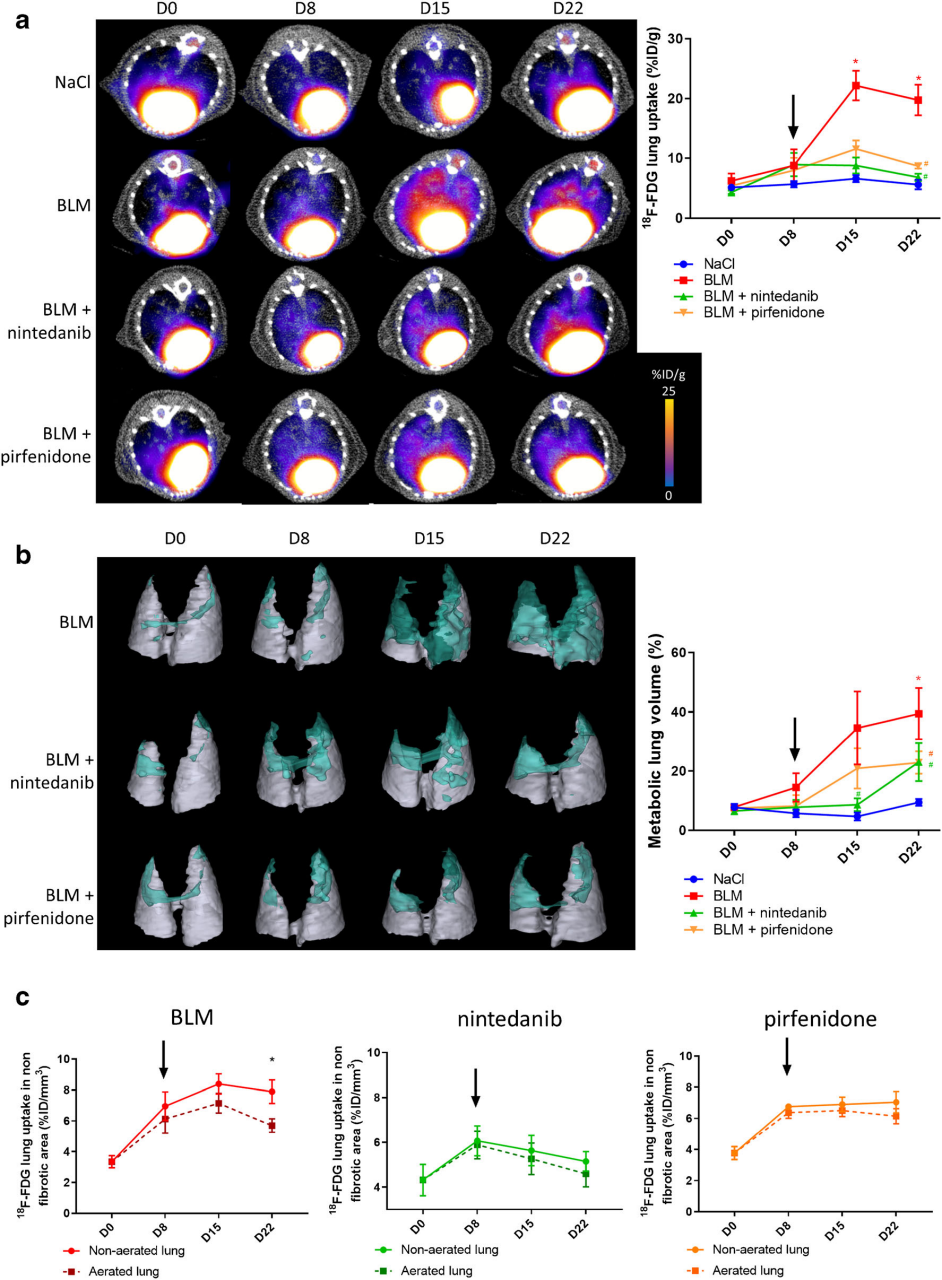
[^18^F]FDG PET can detect advanced lung fibrosis and is suitable for anti-fibrotic therapies follow-up. **a** Representative [^18^F]FDG PET/CT images of NaCl- and BLM-receiving mice treated or not with nintedanib or pirfenidone at D0, D8, D15 and D22. Graph represents evolution of [^18^F]FDG lung uptake (%IG/g) at all time points. Results are presented as mean ± SEM, *n* = 4 for NaCl and *n* = 5 for other groups. Stars (*) are representative of statistical comparison between time points for each group and hashes (#) are representative of statistical comparison between the groups at each time points. *^(#)^*p* < 0.05. Black arrow represents the start of treatments. **b** Representative 3D reconstruction of lung 3DROI segmentation of PET images representative of metabolic lung volume (MLV) of BLM-receiving mice treated or not with nintedanib or pirfenidone at D0, D8, D15 and D22. Cyan represents areas with high [^18^F]FDG lung uptake (above threshold of MLV_threshold_ = (SUVmean)_D0_ + 2SD); grey represents areas with low [^18^F]FDG lung uptake (below the threshold). Graph represents the percentage of evolution of MLV. Results are presented as mean ± SEM, *n* = 4 for NaCl and *n* = 5 for other groups. Stars (*) are representative of statistical comparison between time points for each group and hashes (#) are representative of statistical comparison between the groups at each time points. *p < 0.05. Black arrow represents the start of treatments. **c** Graph represents the evolution of [^18^F]FDG lung uptake in %ID/g of BLM-receiving mice treated or not with nintedanib or pirfenidone at D0, D8, D15 and D22in aerated and non-aerated lung areas (segmented on CT images). Results are presented as mean ± SEM, *n* = 4 for NaCl and *n* = 5 for other groups, *p < 0.05. Black arrow represents the start of treatments

Unlike CT scans, lung uptake of [^18^F]FDG did not increase at early stage (D8) in BLM-receiving mice while it significantly increased at D15 and D22 (Fig. 5a, Suppl Fig. 6a). Pirfenidone and nintedanib treatments dramatically decreased [^18^F]FDG lung uptake at D15 and D22 (Fig. 5a, Suppl Fig. 6a). Similarly, MLV significantly increased in BLM-receiving mice and was decreased by pirfenidone and nintedanib treatment (Fig. 5b, Suppl Fig. 6b). In BLM-receiving mice, the [^18^F]FDG lung uptake was mainly observed in non-aerated lung areas with a significant difference at D22 suggesting that [^18^F]FDG uptake specifically occurs in fibrotic lung areas (Fig. 5c). In parallel, in mice treated with pirfenidone and nintedanib, there was no difference in [^18^F]FDG uptake between aerated and non-aerated lung areas (Fig. 5c). Immunohistofluorescence performed at D23 demonstrated an increase in α-SMA and GLUT-1 expression in BLM-treated lungs which was inhibited by pirfenidone and nintedanib (Suppl Fig. 7a).

#### [^18^F]FMISO PET can detect early lung fibrosis and is suitable for anti-fibrotic therapies follow-up

**Fig. 6:**
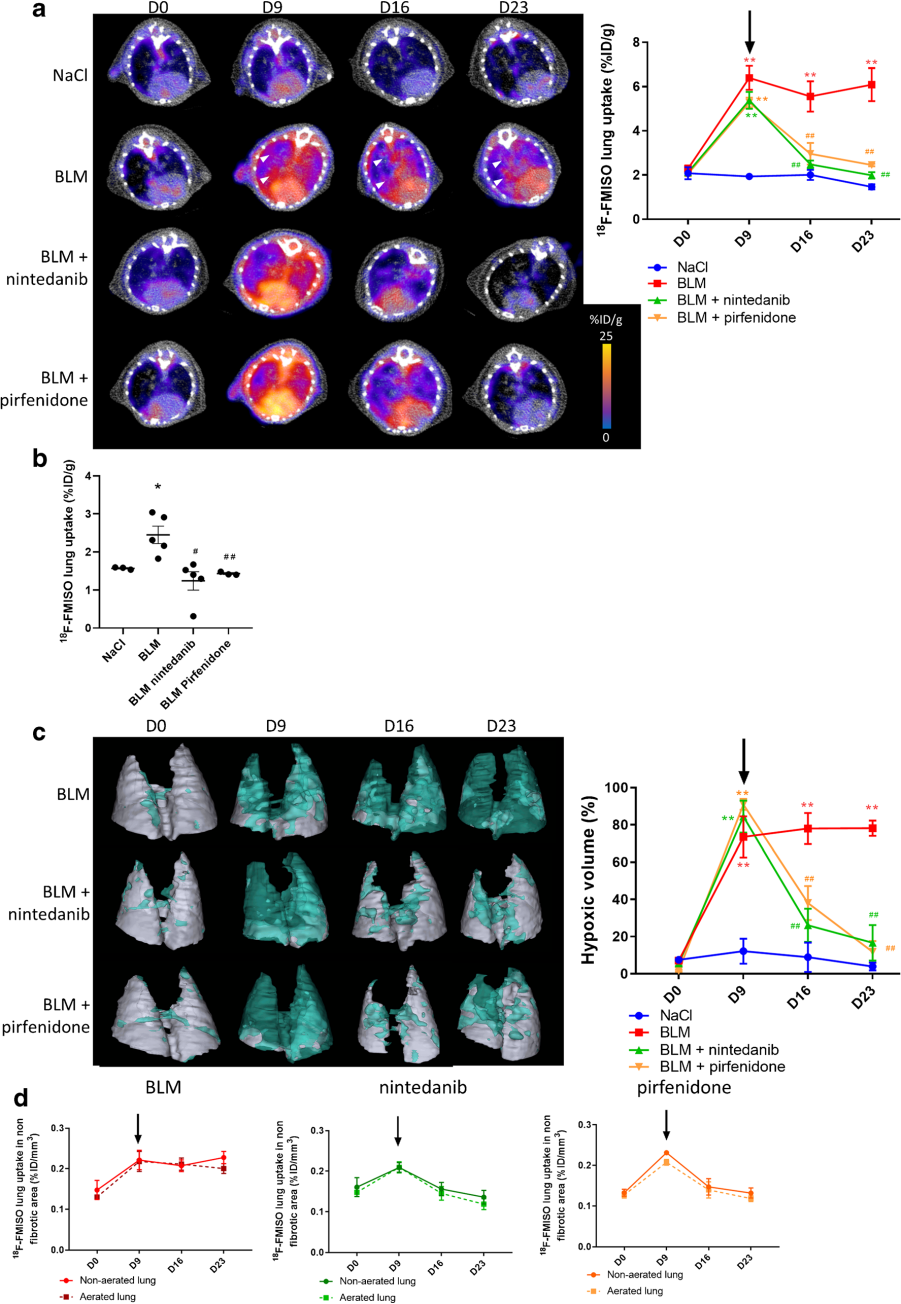
[^18^F]FMISO PET can detect advanced lung fibrosis and is suitable for anti-fibrotic therapies follow-up. **a** Representative [^18^F]FMISO PET/CT images of NaCl- and BLM-receiving mice treated or not with nintedanib or pirfenidone at D0, D9, D16 and D23. Graph represents evolution of [^18^F]FMISO lung uptake (%IG/g) at all time points. Results are presented as mean ± SEM, *n* = 4 for NaCl and *n* = 5 for other groups. Stars (*) are representative of statistical comparison between time points for each group and hashes (#) are representative of statistical comparison between the groups at each time points. **^(##)^*p* < 0.01. Black arrow represents the start of treatments. **b** Radioactivity content in lungs of NaCl- and BLM-receiving mice treated or not with nintedanib or pirfenidone and lung to blood ratio at D23 measured ex vivo with a γ-counter. Results are presented as mean ± SEM, *n* = 4 for NaCl and *n* = 5 for other groups. Stars (*) are representative of comparison of each group with NaCl group and hashes (#) are representative of statistical comparison of each group with the BLM group, *^(#)^*p* < 0.05 and ^##^*p* < 0.01. **c** Representative 3D reconstruction of lung 3DROI segmentation of PET images representative of hypoxic lung volume (HLV) of BLM-receiving mice treated or not with nintedanib or pirfenidone at D0, D9, D16 and D23. Cyan represents areas with high [^18^F]FMISO lung uptake (above threshold of HLV_threshold_ = TBR × 1.4); grey represents areas with low [^18^F]FMISO lung uptake (below the threshold). Graph represents the percentage of evolution of HLV. Results are presented as mean ± SEM, *n* = 4 for NaCl and *n* = 5 for other groups. Stars (*) are representative of statistical comparison between time points for each group and hashes (#) are representative of statistical comparison between the groups at each time points. **^(##)^*p* < 0.01. Black arrow represents the start of treatments. **d** Graph represents the evolution of [^18^F]FMISO lung uptake in %ID/g of BLM-receiving mice treated or not with nintedanib or pirfenidone at D0, D9, D16 and D23 in aerated and non-aerated lung areas (segmented on CT images). Results are presented as mean ± SEM, *n* = 4 for NaCl and *n* = 5 for other groups. Black arrow represents the start of treatments

Lung uptake of [^18^F]FMISO increased at early stage (D9) in BLM-receiving mice and remained higher compared to control up to D23 (Fig. 6a, Suppl Fig. 8a). Interestingly, in BLM-receiving mice, pirfenidone and nintedanib treatments dramatically decreased [^18^F]FMISO lung uptake at D16 and D23 (Fig. 6a, Suppl Fig. 8a). These results were confirmed by gamma counting at D23 in which [^18^F]FMISO lung uptake was significantly higher in BLM-receiving mice and was decreased by nintedanib and pirfenidone (Fig. 6b). Similarly, LHV significantly increased from D9 to D23 in BLM-receiving mice and was decreased by pirfenidone and nintedanib treatments (Fig. 6c, Suppl Fig. 8b). In BLM-receiving mice, the [^18^F]FMISO lung uptake was observed in both aerated and non-aerated lung areas suggesting that [^18^F]FMISO uptake occurs in normal and fibrotic lung areas (Fig. 6d). In BLM-receiving mice, some aerated areas (normal lung density on CT) which showed an uptake of [^18^F]FMISO at D9 (Fig. 6a, white arrowheads) also showed fibrotic consolidations at D16 and D23 demonstrating that early [^18^F]FMISO uptake was indicative of the development of fibrotic lesions (Fig. 6a, white arrowheads). Immunohistochemistry performed at D23 demonstrated an increase in HIF-1 expression in BLM-treated lungs which was inhibited by pirfenidone and nintedanib (Suppl Fig. 9a).

#### [^18^F]FMISO PET is correlated with fibrosis progression and nintedanib efficacy

**Fig. 7:**
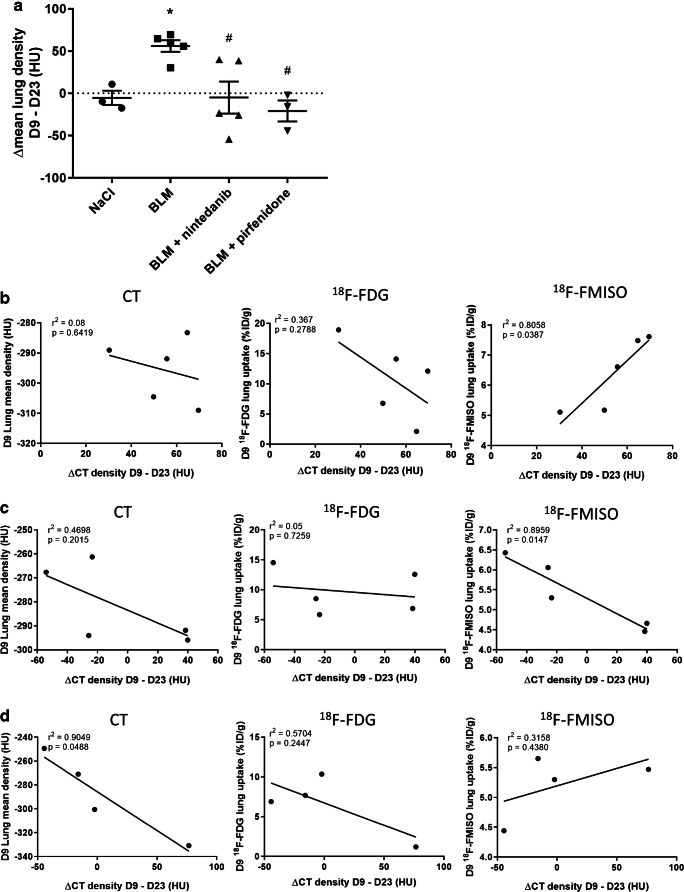
[^18^F]FMISO PET is correlated with fibrosis progression and nintedanib efficacy. **a** Variation of mean lung density on CT scans of NaCl- and BLM-receiving mice treated or not with nintedanib or pirfenidone between D9 and D23 (ΔCT^D9-D23^). Results are presented as mean ± SEM, *n* = 4 for NaCl and *n* = 5 for other groups. Stars (*) are representative of comparison of each group with the NaCl group and hashes (#) are representative of statistical comparison of each group with the BLM group, *^(#)^*p* < 0.05. **b** Correlation between, D9 mean lung density (left), D9 [^18^F]FDG lung uptake (center) or D9 [^18^F]FMISO lung uptake (right) and ΔCT^D9-D23^ in BLM-receiving mice. **c** Correlation between, D9 mean lung density (left), D9 [^18^F]FDG lung uptake (center) or D9 [^18^F]FMISO lung uptake (right) and ΔCT^D9-D23^ in BLM-receiving mice treated with nintedanib. **d** Correlation between, D9 mean lung density (left), D9 [^18^F]FDG lung uptake (center) or D9 [^18^F]FMISO lung uptake (right) and ΔCT^D9-D23^ in BLM-receiving mice treated with pirfenidone

Fibrosis progression as well as pirfenidone and nintedanib efficacy was quantified using the variation of MLD between D9 and D23 (ΔCT^D9-D23^). BLM induced an increase in ΔCT^D9-D23^ demonstrating fibrosis progression between D9 and D23 (Fig. 7a). Both nintedanib and pirfenidone significantly decreased ΔCT^D9-D23^ showing therapy efficacy (Fig. 7a). In order to determine the predictive value of early CT imaging, [^18^F]FDG PET and [^18^F]FMISO PET, correlation between ΔCT^D9-D23^ and values at D9 of MLD, [^18^F]FDG uptake and [^18^F]FMISO uptake were performed. Interestingly, in BLM-receiving mice, [^18^F]FMISO uptake at D9 positively correlated with ΔCT^D9-D23^ showing that lungs with higher [^18^F]FMISO uptake at D9 were those with higher fibrosis progression (Fig. 7b). On the contrary, MLD and [^18^F]FDG uptake at D9 did not correlate with ΔCT^D9-D23^ (Fig. 7b).

Similarly, [^18^F]FMISO uptake at D9 positively correlated with ΔCT^D9-D23^ of nintedanib-treated mice demonstrating that lungs with higher [^18^F]FMISO uptake at D9 were those in which nintedanib showed the best efficacy (Fig. 7c). On the contrary, MLD and [^18^F]FDG uptake at D9 did not correlate with ΔCT^D9-D23^ in nintedanib-treated animals (Fig. 7c).

Neither [^18^F]FDG uptake nor [^18^F]FMISO uptake significantly correlated with ΔCT^D9-D23^ of pirfenidone-treated mice demonstrating that the early uptake of these two tracers is not correlated with pirfenidone efficacy (Fig. 7d). On the contrary, MLD at D9 negatively correlated with ΔCT^D9-D23^ of pirfenidone-treated mice meaning that pirfenidone showed the best efficacy on lungs with higher MLD at early fibrotic stage (D9, Fig. 7d).

Similar results were obtained when correlating early (D9) CT, [^18^F]FDG and [^18^F]FMISO uptakes with the amount of collagen at D23 measured by histological staining. Only early [^18^F]FMISO uptake significantly and positively correlated with the amount of collagen at D23 in BLM-receiving mice demonstrating that the lung with high early [^18^F]FMISO uptake had the most fibrotic outcome (Suppl Fig. 10a). Similarly, only early [^18^F]FMISO uptake significantly and negatively correlated with end collagen content in BLM-receiving mice treated with nintedanib demonstrating that the lung with high early [^18^F]FMISO uptake responded better to nintedanib (Suppl Fig. 10b). Neither early (D9) CT, [^18^F]FDG nor [^18^F]FMISO uptakes correlated with response to pirfenidone (Suppl Fig. 10c).

## Discussion

In vivo molecular imaging has become a central tool in preclinical research, clinical trials and medical practice for the diagnosis and monitoring of several diseases. Imaging of IPF remains limited to HRCT in the clinical practice both for diagnosis and monitoring. In the current study, we confirm in a preclinical model that longitudinal CT imaging is a reliable tool to monitor the severity and progression of lung fibrotic areas. Semi-automatic mice lung segmentation allowed accurate CT quantification with parameters such as MLD and the determination of aerated lung areas which are decreased during fibrosis. In our study, CT quantification detected fibrotic changes in the lungs of BLM-receiving mice from D9 with progression up to D23 in correlation with classical histological determination of lung collagen content in this preclinical model. Our results are in accordance with previous studies demonstrating that lung CT imaging segmentation and quantification using equivalent methods were in good agreement with histological read-outs and were able to quantify effectively and non-invasively disease progression longitudinally in several lung fibrosis models [21–23]. Furthermore, we demonstrate that our CT imaging lung segmentation/quantification allowed the monitoring of pirfenidone and nintedanib anti-fibrotic efficacy by reducing MLD and increasing lung aerated areas from D9 up to D23. Similar results were found in a study monitoring the impact of imatinib on aerated lung volume and MLD by longitudinal CT scans demonstrating the usefulness of such technique to monitor therapy efficacy in fibrosis animal models [24].

The main clinical challenge remains the impossibility to predict the disease course at the time of diagnosis. Cocconcelli et al. [25] demonstrated that fibrotic extent in HRCT of IPF patients was useful to differentiate rapid from slow progressors at presentation. In addition, early structural changes on HRCT have been shown to predict progression in lung function of IPF patients [26] and HRCT scanning indexes including MLD represent additive value for prediction of outcome [27]. On the contrary, our preclinical study on BLM-induced lung fibrosis in mice did not demonstrate a correlation between early CT quantification and disease progression. This discrepancy may be explained by the differences between the lesions observable on CT in animal models and in human IPF. While BLM-induced fibrosis remains a robust model regrouping characteristic features such as collagen accumulation and deterioration of lung function, it does not fully recapitulate IPF features such as honeycombing which is a hallmark of HRCT in IPF patients. Another bias resides in the progression pattern of BLM-induced fibrosis which may be more homogeneous compared with one of IPF patients due to controlled experimental procedures. In addition, baseline HRCT in IPF patients are likely representative of more advanced lung fibrosis than in our animal model due to diagnosis delays. As a consequence, early fibrotic lesions observed in our model at D9 may not be enough advanced to correlate with fibrosis progression as observed in human IPF. Nevertheless, our preclinical study highlighted the fact that baseline CT quantification correlated with pirfenidone efficacy in accordance with a clinical study by Higo et al. [28] which demonstrates that an early deterioration of HRCT was significantly more observed in the progressive versus improved/stable IPF patients under pirfenidone treatment. Similarly, increased honeycombing and ground glass opacities on HRCT were shown to be associated with progressive IPF patients under pirfenidone [29]. Despite such promising preclinical and clinical results, HRCT quantification is not yet integrated in a routine clinic.

Our results confirm the upregulation of Glut-1 specifically in α-SMA positive myofibroblasts in BLM-induced fibrotic lungs. Accordingly, [^18^F]FDG lung uptake was specifically found in fibrotic areas and was only increased at later stages from D15 suggesting that it requires a certain amount of differentiated myofibroblasts to be observable. Our results are in accordance with the previous finding in preclinical models showing an increase in [^18^F]FDG lung uptake upon BLM at D15 and clinical findings showing that lung areas with high [^18^F]FDG uptake were associated with regions of lung parenchymal changes on HRCT [5–7]. The temporal and spatial distribution of the [^18^F]FDG uptake in the fibrotic lung both in clinical and preclinical setting suggest that [^18^F]FDG PET may detect fibrotic regions that are already visible on HRCT, thus limiting its interest as a novel imaging tool for lung fibrosis. In addition, a number of issues persist with the use of [^18^F]FDG for IPF management. Even though glucose transporters are upregulated on myofibroblasts in IPF, macrophages and cancer cells are also characterized by an increased uptake of [^18^F]FDG which can lead to misinterpretation of [^18^F]FDG PET imaging in IPF patients with common co-morbidities such as lung infection or lung cancer. Interestingly, in line with our results, a recent study demonstrated the inefficiency of [^18^F]FDG PET imaging to assess the early response of IPF patients to nintedanib or pirfenidone [18], further highlighting the crucial need for a better tracer for IPF early detection and therapy efficacy monitoring.

Chronic hypoxia of the lung is a significant clinical feature of patients with IPF and hypoxia exposure exacerbates bleomycin-induced lung fibrosis [15]. Interestingly, Burman et al. [15] demonstrated that BLM induced the formation of hypoxic patches localized around fibrotic areas. Furthermore, pimonidazole, a compound which specifically accumulates in cells in hypoxic conditions with a similar mechanism than [^18^F]FMISO, was found to accumulate in alveolar epithelial cells boarding fibrotic lung areas upon bleomycin in mice. Our data support the fact that a hypoxic microenvironment develops in the fibrotic lungs upon BLM in mice with the upregulation of hypoxic markers such as HIF-1α associated with an increase in [^18^F]FMISO lung uptake in correlation with fibrosis severity, as already demonstrated in oncology [30]. We demonstrate that the increase in [^18^F]FMISO lung uptake occurs in the area appearing “normal” on early CT scans and which will evolve towards fibrotic lesion. These data are in line with other studies demonstrating that HIF-1α and CAIX were upregulated not only in areas of active fibrosis but also within areas of IPF lung that appear histologically normal [11]. These findings suggest that activation of hypoxia signalling is driving the remodelling of areas in the IPF lung which are not yet fibrotic thus promoting disease progression. These data are also supported by the fact that [^18^F]FMISO uptake occurred at early stage from D9 suggesting the hypoxia imaging with [^18^F]FMISO could represent an early marker of fibrosis progression. While these preclinical results are certainly promising, their relevance for human IPF may not be unchallenging. BLM-induced fibrosis, while a very robust model of progressive lung fibrosis, may show some important limitations for clinical translation regarding hypoxia signalling. As demonstrated in our study, BLM induces a diffuse and massive expression of HIF1α in mice lungs which is rather different from HIF1α signal in IPF lung in which HIF1α expression has been shown to be more restricted to hyperplastic alveolar epithelial cells and not (myo)fibroblasts [10, 15, 31]. As a consequence, our preclinical results certainly need to be further confirmed in phase I clinical studies evaluating the relevance of [^18^F]FMISO PET in IPF patients.

In addition, we demonstrate that early [^18^F]FMISO lung uptake correlates with fibrosis progression. Upon BLM, mice lungs with higher [^18^F]FMISO uptake show the most severe fibrosis progression. Similarly, unlike CT scans and [^18^F]FDG, [^18^F]FMISO imaging correlates with nintedanib efficacy. Upon BLM, mice lungs with higher [^18^F]FMISO uptake show a better anti-fibrotic response with nintedanib. Fibrosis progression and therapy efficacy have been measured by the variation of MLD between start and end of treatment (D9 and D23) in BLM-receiving mice treated or not with pirfenidone and nintedanib. This method therefore assumes that CT scan is an accurate tool to quantify lung fibrosis. In addition, as mentioned before, differences in CT scans between preclinical model and human IPF represent a limitation regarding the translation of our results in clinical setting. To further validate our approach, we demonstrated that early [^18^F]FMISO lung uptake also correlated with end collagen content which is a validated readout in preclinical lung fibrosis studies. Our preclinical data are in line with recent clinical studies showing that intracellular hypoxia measured by [^18^F]FMISO PET has prognostic impact in patients with breast and pancreas cancer [32, 33]. Moreover, Quintela-Fandino et al. [34] demonstrated a predictive role of [^18^F]FMISO PET in HER2-negative early breast cancer treated with nintedanib, detecting the patients that did not experience benefit from it based on baseline tumour oxygenation levels and further supporting the potential impact of hypoxia imaging as an early marker of nintedanib efficacy.

## Conclusion

In light of the lack of tracers to monitor lung fibrosis, our study proposes to evaluate the impact of [^18^F]FDG PET and [^18^F]FMISO PET on lung fibrosis monitoring in a preclinical model. While both tracers are able to detect BLM-induced lung fibrosis in mice as well as CT (Suppl Fig. 11a), [^18^F]FMISO PET allows an earlier detection of the disease than [^18^F]FDG PET which may be crucial with a view to promoting early diagnosis in lung fibrosis. In addition, early [^18^F]FMISO lung uptake appears to be a better indicator of BLM-induced lung fibrosis aggressiveness and response to nintedanib. As a consequence, [^18^F]FMISO PET may be an interesting tool to evaluate as a first step towards personalized medicine in IPF to identify patients likely to rapidly/dramatically progress and likely to respond to nintedanib rather than pirfenidone. It would therefore become an excellent tool to drive therapeutic decision in order to ultimately improve patients’ outcome in this devastating disease. To that end, there is an urgent need to provide phase I clinical studies evaluating the relevance of [^18^F]FMISO PET in IPF patients.

## Supplementary information

Supplemental figure 1. a/ Schematic representation of the design of our pilot study. b/ Schematic representation of the design of our longitudinal study. (PNG 419 kb)

High resolution image (TIF 602 kb)

Supplemental figure 2. a/ 18F-FDG lung uptake in SUVmean (left), SUVmax (center) and lung-to-backgroud ratio (right) in Nacl- and BLM-receiving mice at D21. Results are presented as mean ± SEM, n = 3 for Nacl and n = 6 for BLM. *p<0.05. b/ Correlation between, mean lung density on CT images and 18F-FDG lung uptake in SUVmean (upper) and SUVmax (lower). (PNG 400 kb)

High resolution image (TIF 1290 kb)

Supplemental figure 3. a/ 18F-FMISO lung uptake in SUVmean (left), SUVmax (center) and lung-to-backgroud ratio (right) in Nacl- and BLM-receiving mice at D21. Results are presented as mean ± SEM, n = 3 for Nacl and n = 6 for BLM. *p<0.05. Black arrow represents the start of treatments. b/ Correlation between, mean lung density on CT images and 18F-FMISO lung uptake in SUVmean (upper) and SUVmax (lower). (PNG 171 kb)

High resolution image (TIF 428 kb)

Supplemental figure 4. a/ Representative fluorescence imaging of HypoxisenseTM 680 of Nacl- and BLM-receiving mice at D21. Sham = non-injected animal. b/ HypoxisenseTM 680 quantification in mean lung radiance efficacy. Results are presented as mean ± SEM, n = 3 for Nacl and n = 6 for BLM. **p<0.05. c/ Correlation between HypoxisenseTM 680 quantification and picrosirius red staining in corresponding lungs. (PNG 652 kb)

High resolution image (TIF 757 kb)

Supplemental figure 5. a/ Representative 3D reconstruction of lung 3DROI segmentation of lung CT images of BLM-receiving mice treated or not with nintedanib of pirfenidone at D0, D9, D16 and D23. Red represents high density lung areas (-100 to 300 HU) representative of non-aerated lungs, gray represents normal density lung areas (-800 to -100 HU) representative of aerated lungs. (PNG 3272 kb)

High resolution image (TIF 11367 kb)

Supplemental figure 6. a/ Graph represents evolution of 18F-FDG lung uptake in Nacl- and BLM- and receiving mice treated or not with nintedanib of pirfenidone at D0, D9, D15 and D22 in SUVmean (left), target to background ratio (center) and SUVmax (right). Results are presented as mean ± SEM, n = 4 for Nacl and n = 5 for other goups. Stars (*) are representative of statistical comparison between time points for each groups and hashs (#) are representative of statistical comparison between the groups at each time points. *(#)p<0.05, **(##)p<0.01. b/ Representative 3D reconstruction of lung 3DROI segmentation of 18F-FDG PET images representative of metabolic lung volume (MLV) of BLM-receiving mice treated or not with nintedanib of pirfenidone at D0, D8, D15 and D22. Cyan represents areas with high 18F-FDG lung uptake (above threshold of MLVthreshold = (SUVmean)D0 + 2SD), gray represents areas with low 18F-FDG lung uptake (below the threshold). (PNG 1786 kb)

High resolution image (TIF 5831 kb)

Supplemental figure 7. a/ Immunofluorescence staining of α-SMA (red) and GLUT-1 (green) on lung section form Nacl- and BLM-receiving mice treated or not with nintedanib of pirfenidone at D21. Scale bars = 100 μm. (PNG 3979 kb)

High resolution image (TIF 14565 kb)

Supplemental figure 8. a/ Graph represents evolution of 18F-FMISO lung uptake in Nacl- and BLM- and receiving mice treated or not with nintedanib of pirfenidone at D0, D9, D15 and D22 in SUVmean (left), target to background ratio (right) and SUVmax (lower). Results are presented as mean ± SEM, n = 4 for Nacl and n = 5 for other goups. Stars (*) are representative of statistical comparison between time points for each groups and hashs (#) are representative of statistical comparison between the groups at each time points. *(#)p<0.05. Black arrow represents the start of treatments. b/ Representative 3D reconstruction of lung 3DROI segmentation of 18F-FMISO PET images representative of hypoxic lung volume (HLV) of BLM-receiving mice treated or not with nintedanib of pirfenidone at D0, D9, D16 and D23. Cyan represents areas with high 18F-FMISO lung uptake (above threshold of HLVthreshold= TBR x 1.4), gray represents areas with low 18F-FMISO lung uptake (below the threshold). (PNG 2265 kb)

High resolution image (TIF 7167 kb)

Supplemental figure 9. a/ HIF-1α staining on lung section form Nacl- and BLM-receiving mice treated or not with nintedanib of pirfenidone at D21. Insert represent the isotype control. Scale bars = 100 μm. (PNG 2551 kb)

High resolution image (TIF 8760 kb)

Supplemental figure 10. a/ Correlation between, D9 mean lung density (left), D9 [18F]FDG lung uptake (center) or D9 [18F]FMISO lung uptake (right) and intensity of picrosirius red staining in BLM-receiving mice. b/ Correlation between, D9 mean lung density (left), D9 [18F]FDG lung uptake (center) or D9 [18F]FMISO lung uptake (right) and intensity of picrosirius red staining in BLM-receiving mice treated with nintedanib. c/ Correlation between, D9 mean lung density (left), D9 [18F]FDG lung uptake (center) or D9 [18F]FMISO lung uptake (right) and intensity of picrosirius red staining in BLM-receiving mice treated with pirfenidone. (PNG 525 kb)

High resolution image (TIF 1605 kb)

Supplemental figure 11. a/ Time course of mean lung density, [18F]FMISO uptake and [18F]FDG uptake variations upon BLM and nintedanib/pirfenidone treatments. (PNG 318 kb)

High resolution image (TIF 951 kb)
