# Supplementary figures and images for: [18F]FMISO PET/CT imaging of hypoxia as a non-invasive biomarker of disease progression and therapy efficacy in a preclinical model of pulmonary fibrosis: comparison with the [18F]FDG PET/CT approach
Source: Eur J Nucl Med Mol Imaging. 2021 Feb 13;48(10):3058–74. doi: 10.1007/s00259-021-05209-2 (PMC8426306; doi:10.1007/s00259-021-05209-2)

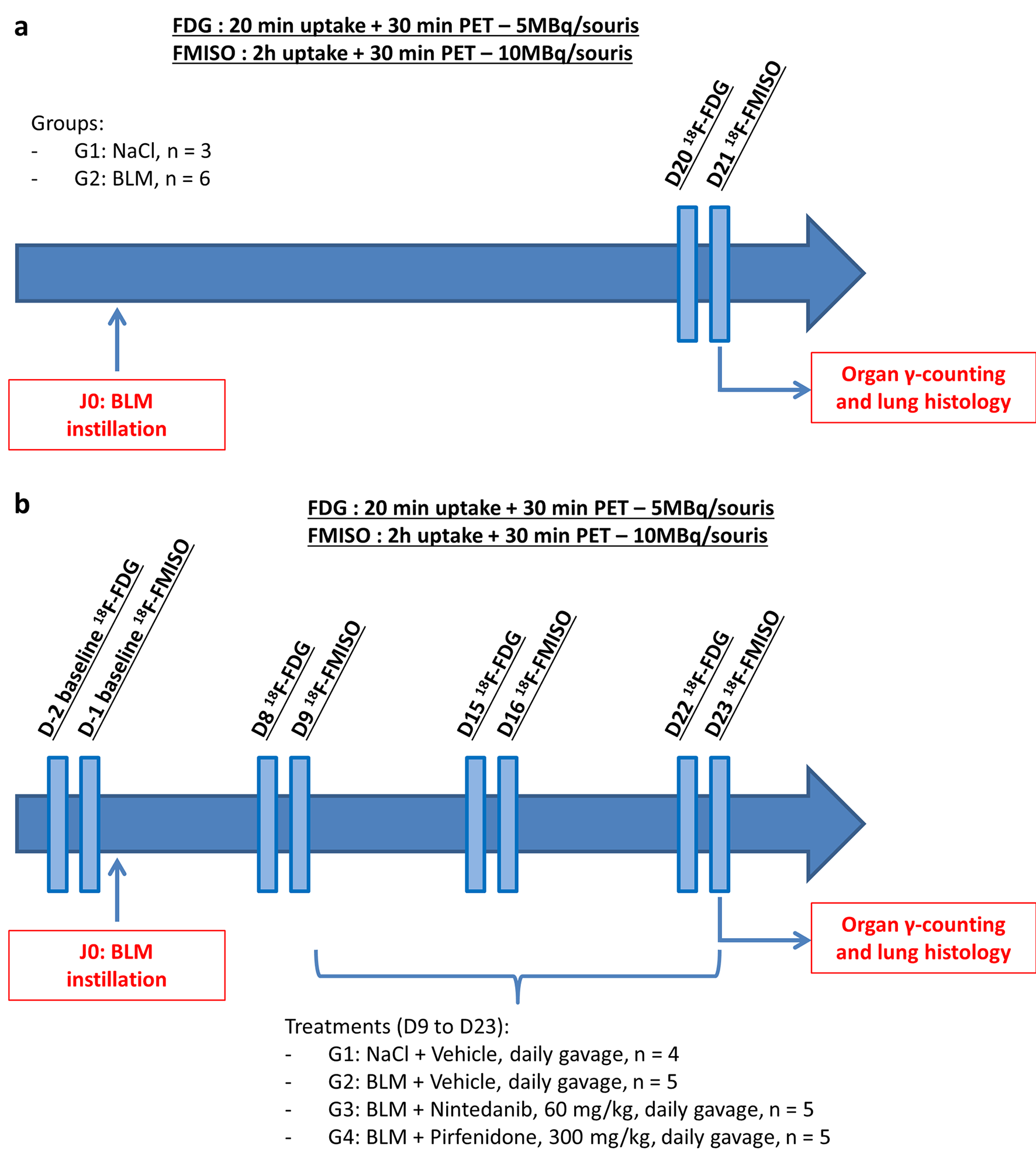

Supplement: Supplementary file 1 — a/ Schematic representation of the design of our pilot study. b/ Schematic representation of the design of our longitudinal study. (PNG 419 kb) [file 259_2021_5209_Fig8_ESM.png]

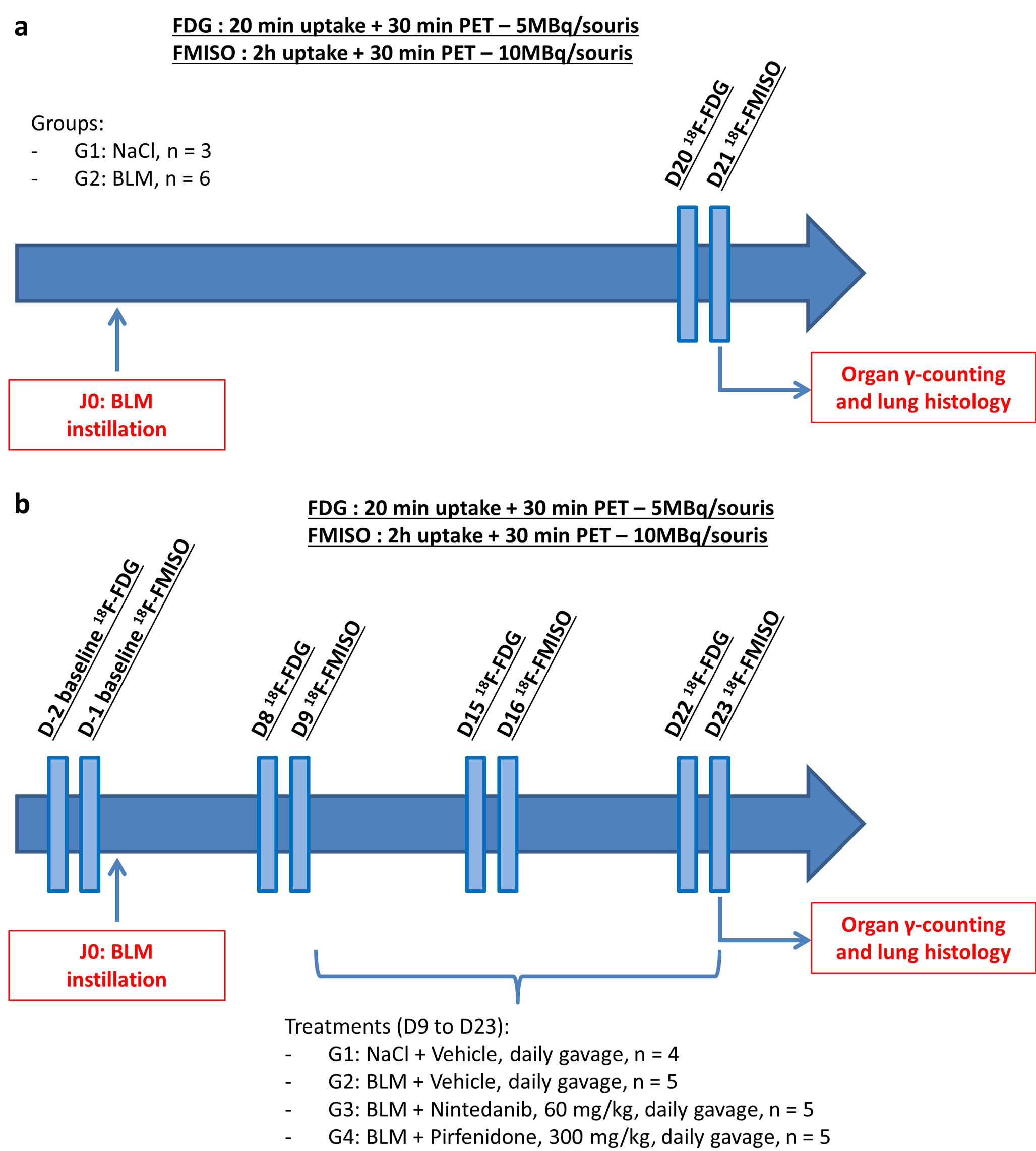

Supplement: Supplementary file 2 — High resolution image (TIF 602 kb) [file 259_2021_5209_MOESM1_ESM.tif]

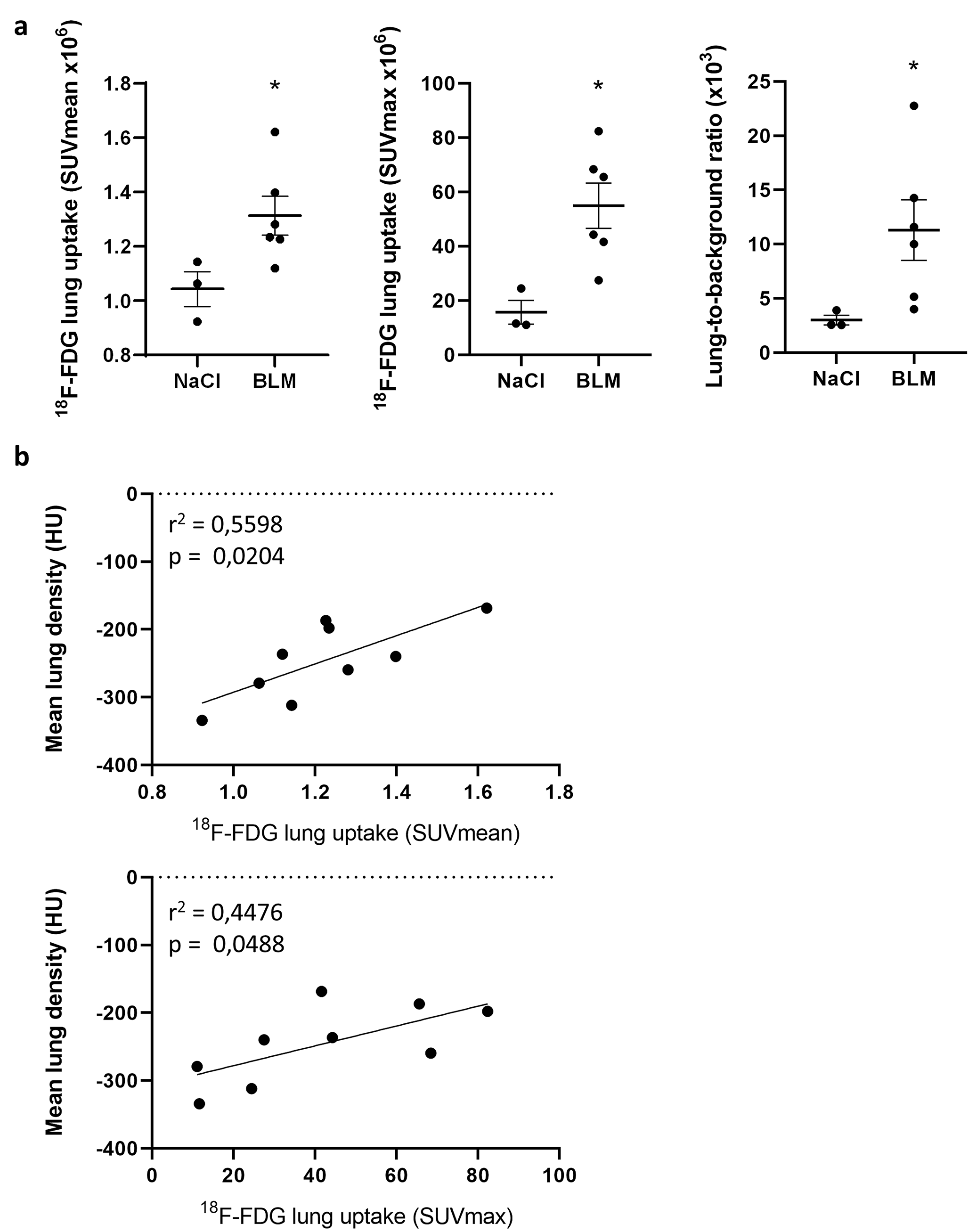

Supplement: Supplementary file 3 — a/ 18F-FDG lung uptake in SUVmean (left), SUVmax (center) and lung-to-backgroud ratio (right) in Nacl- and BLM-receiving mice at D21. Results are presented as mean ± SEM, n = 3 for Nacl and n = 6 for BLM. *p<0.05. b/ Correlation between, mean lung density on CT images and 18F-FDG lung uptake in SUVmean (upper) and SUVmax (lower). (PNG 400 kb) [file 259_2021_5209_Fig9_ESM.png]

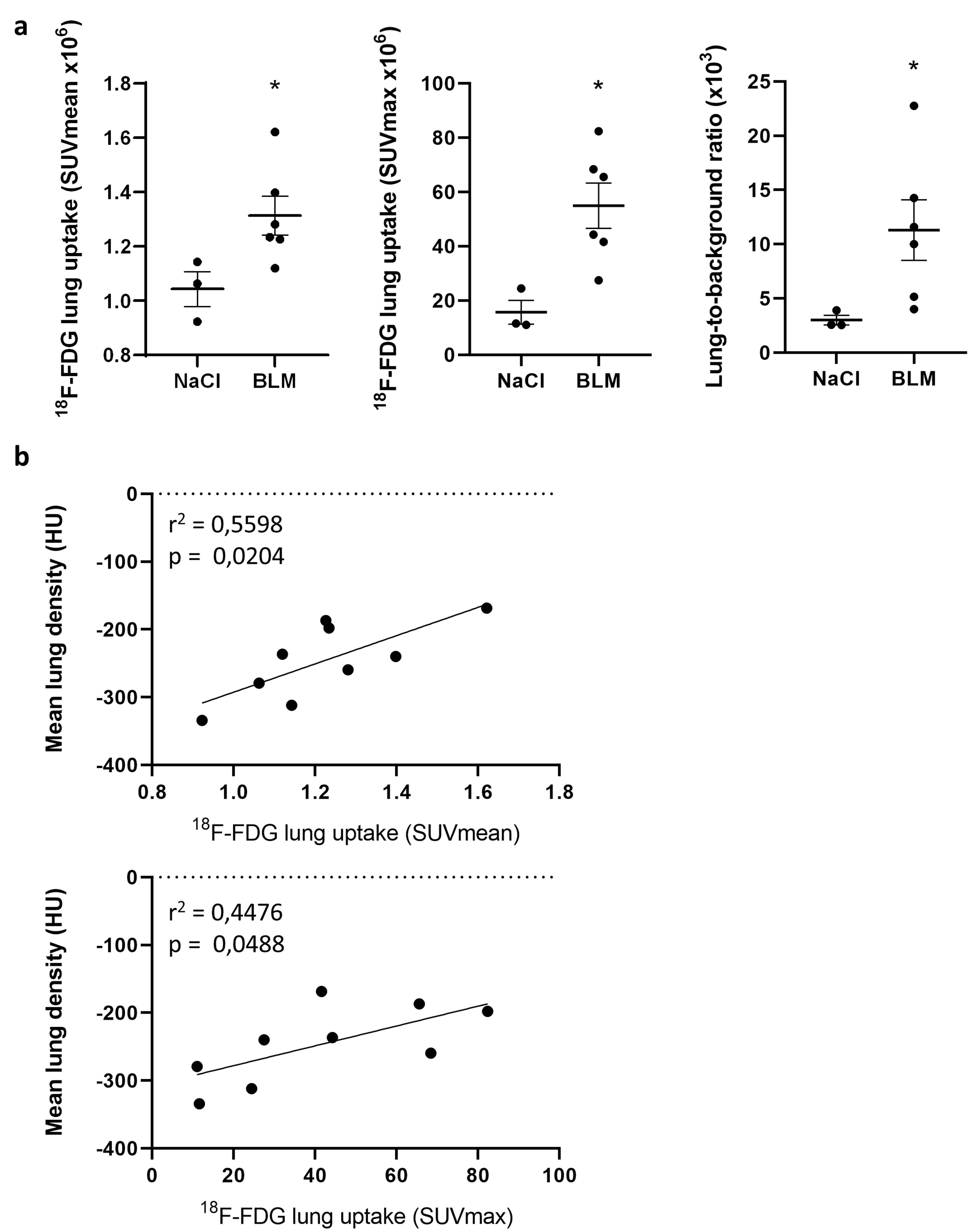

Supplement: Supplementary file 4 — High resolution image (TIF 1290 kb) [file 259_2021_5209_MOESM2_ESM.tif]

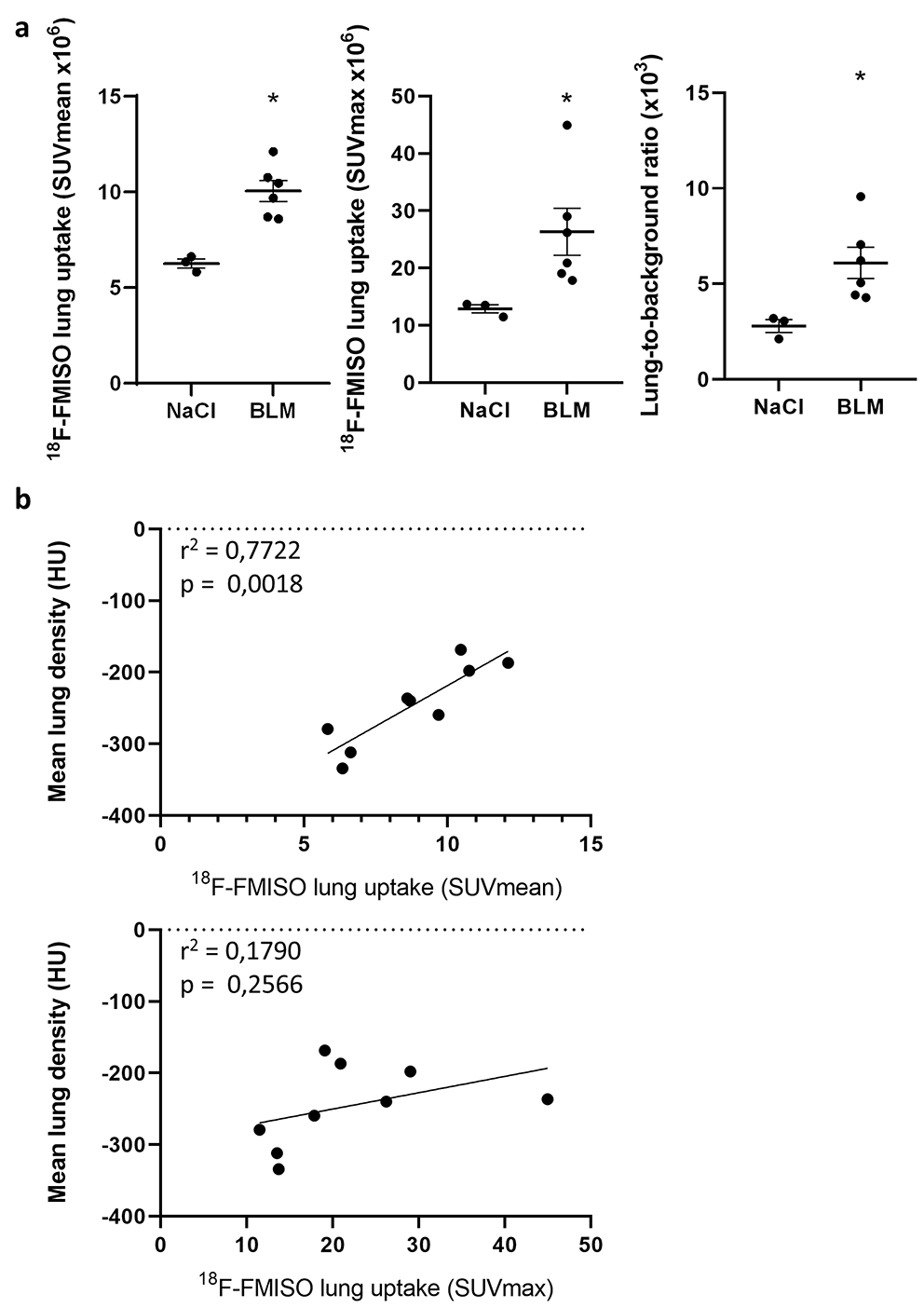

Supplement: Supplementary file 5 — a/ 18F-FMISO lung uptake in SUVmean (left), SUVmax (center) and lung-to-backgroud ratio (right) in Nacl- and BLM-receiving mice at D21. Results are presented as mean ± SEM, n = 3 for Nacl and n = 6 for BLM. *p<0.05. Black arrow represents the start of treatments. b/ Correlation between, mean lung density on CT images and 18F-FMISO lung uptake in SUVmean (upper) and SUVmax (lower). (PNG 171 kb) [file 259_2021_5209_Fig10_ESM.png]

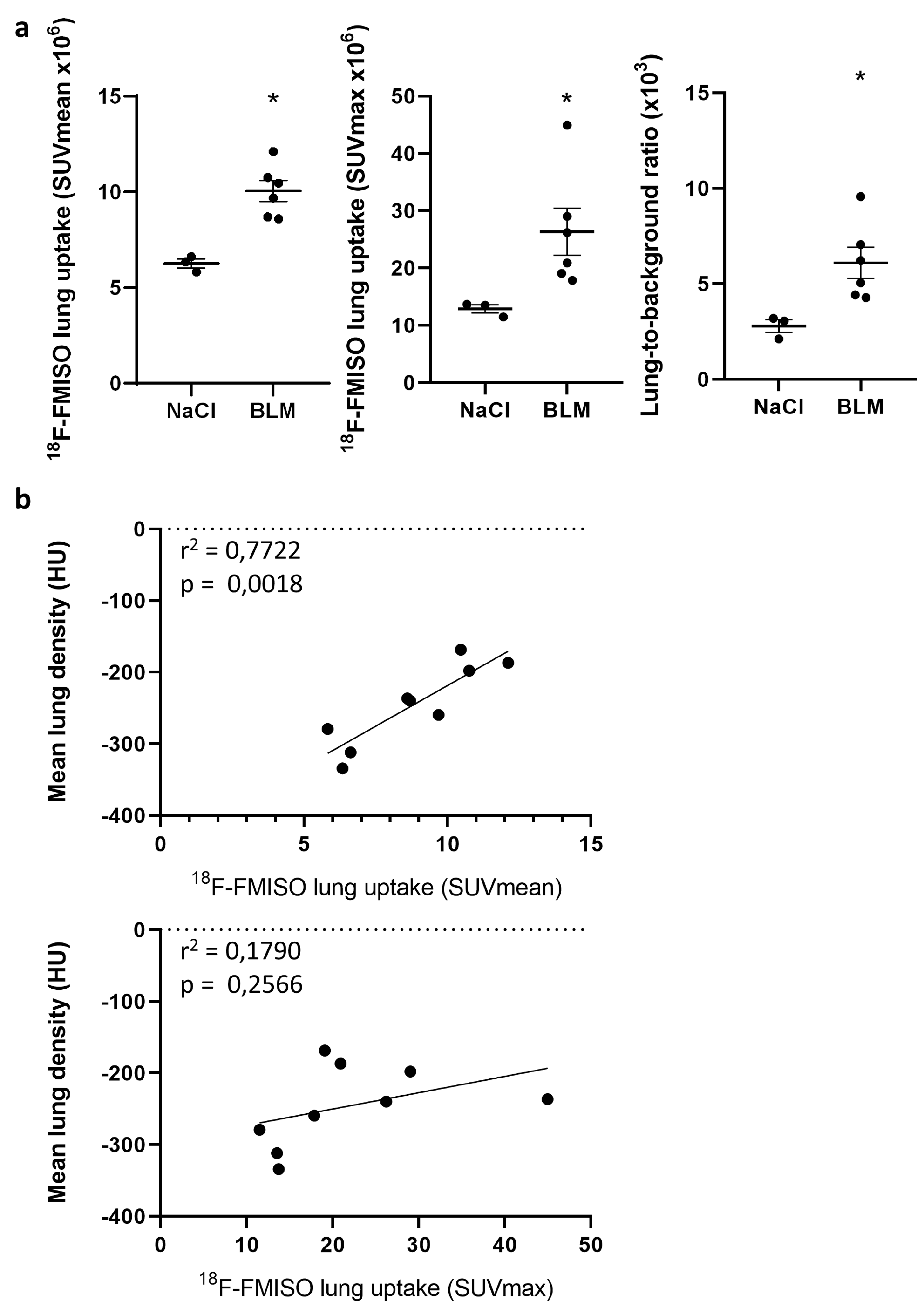

Supplement: Supplementary file 6 — High resolution image (TIF 428 kb) [file 259_2021_5209_MOESM3_ESM.tif]

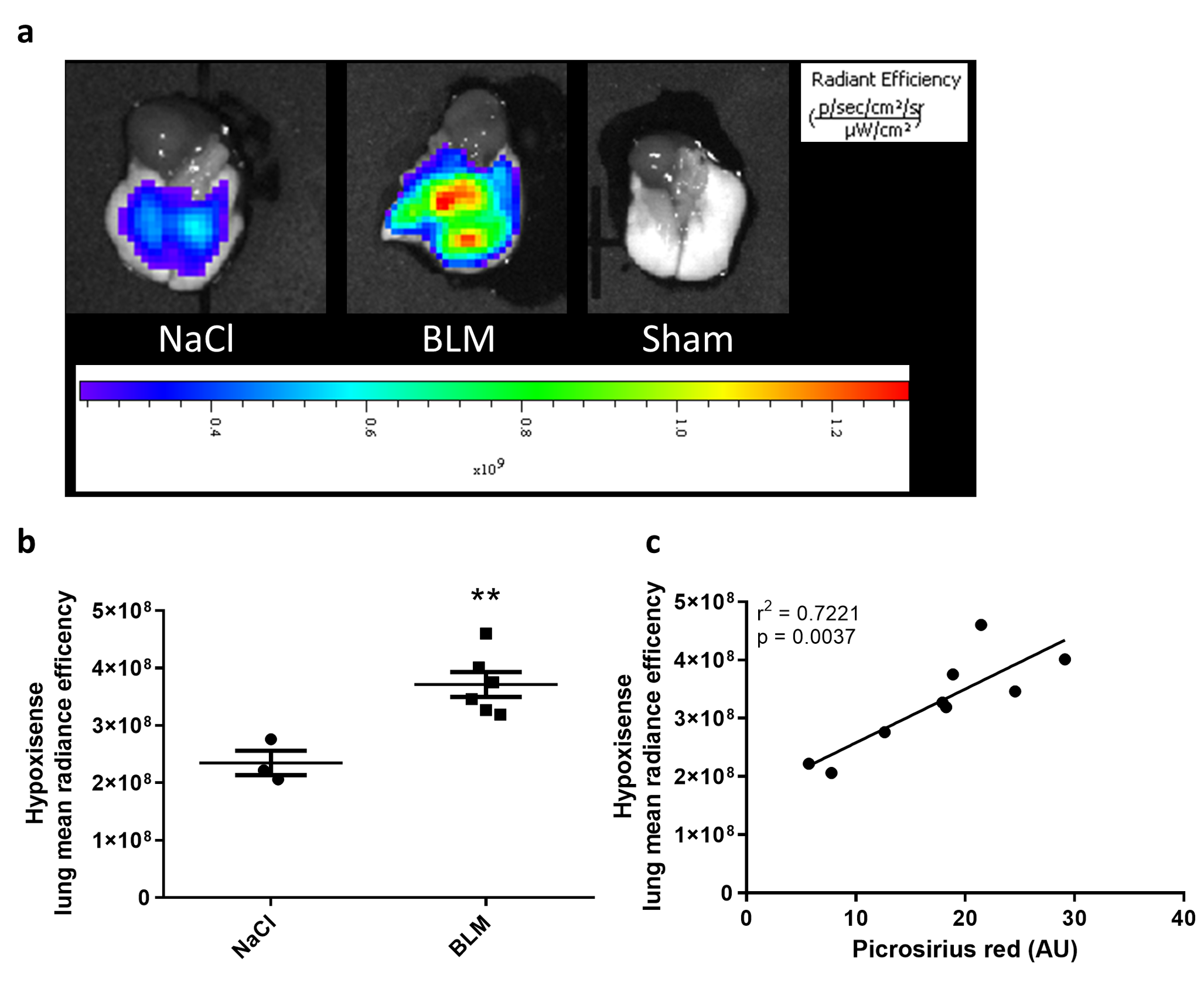

Supplement: Supplementary file 7 — a/ Representative fluorescence imaging of HypoxisenseTM 680 of Nacl- and BLM-receiving mice at D21. Sham = non-injected animal. b/ HypoxisenseTM 680 quantification in mean lung radiance efficacy. Results are presented as mean ± SEM, n = 3 for Nacl and n = 6 for BLM. **p<0.05. c/ Correlation between HypoxisenseTM 680 quantification and picrosirius red staining in corresponding lungs. (PNG 652 kb) [file 259_2021_5209_Fig11_ESM.png]

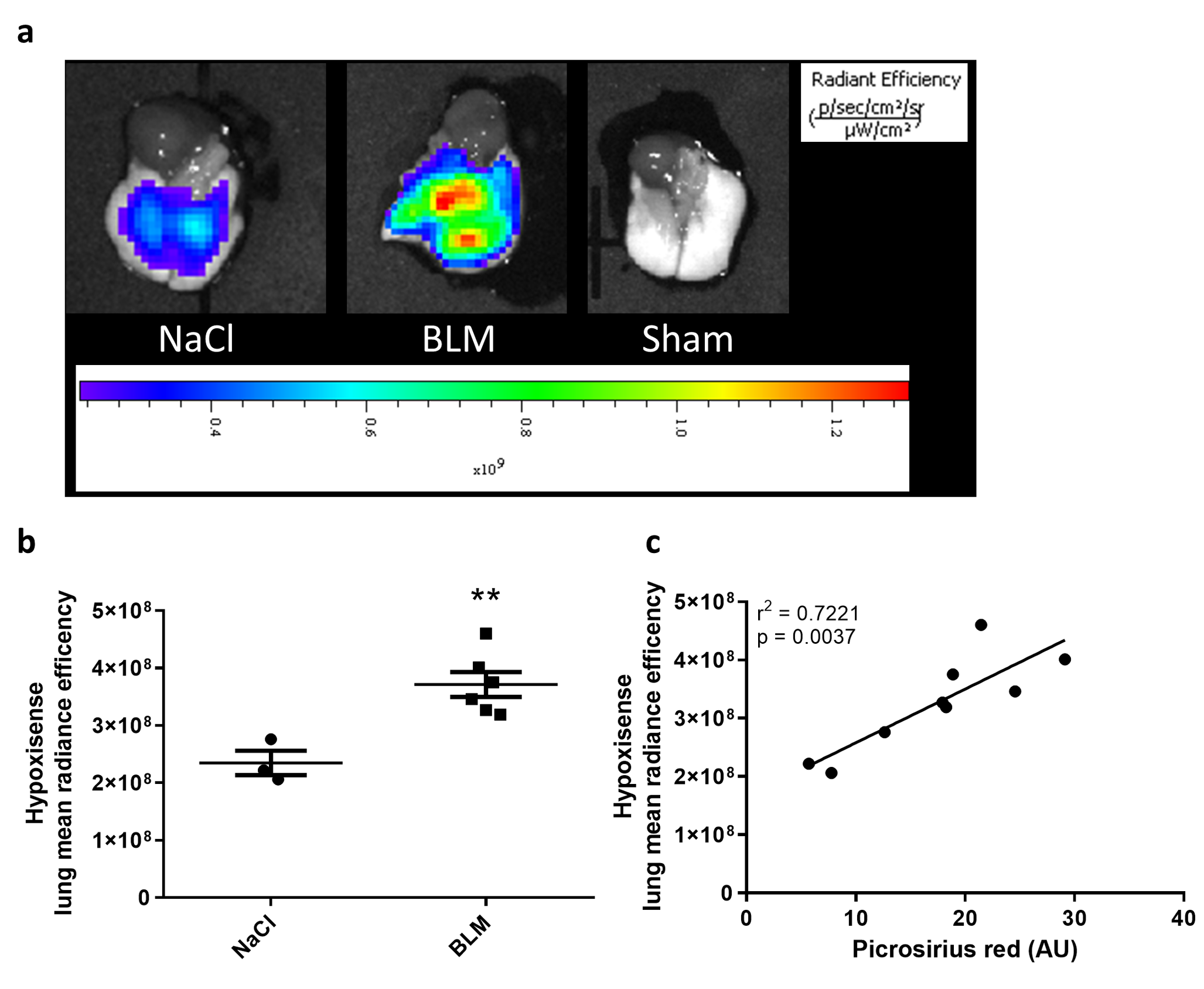

Supplement: Supplementary file 8 — High resolution image (TIF 757 kb) [file 259_2021_5209_MOESM4_ESM.tif]

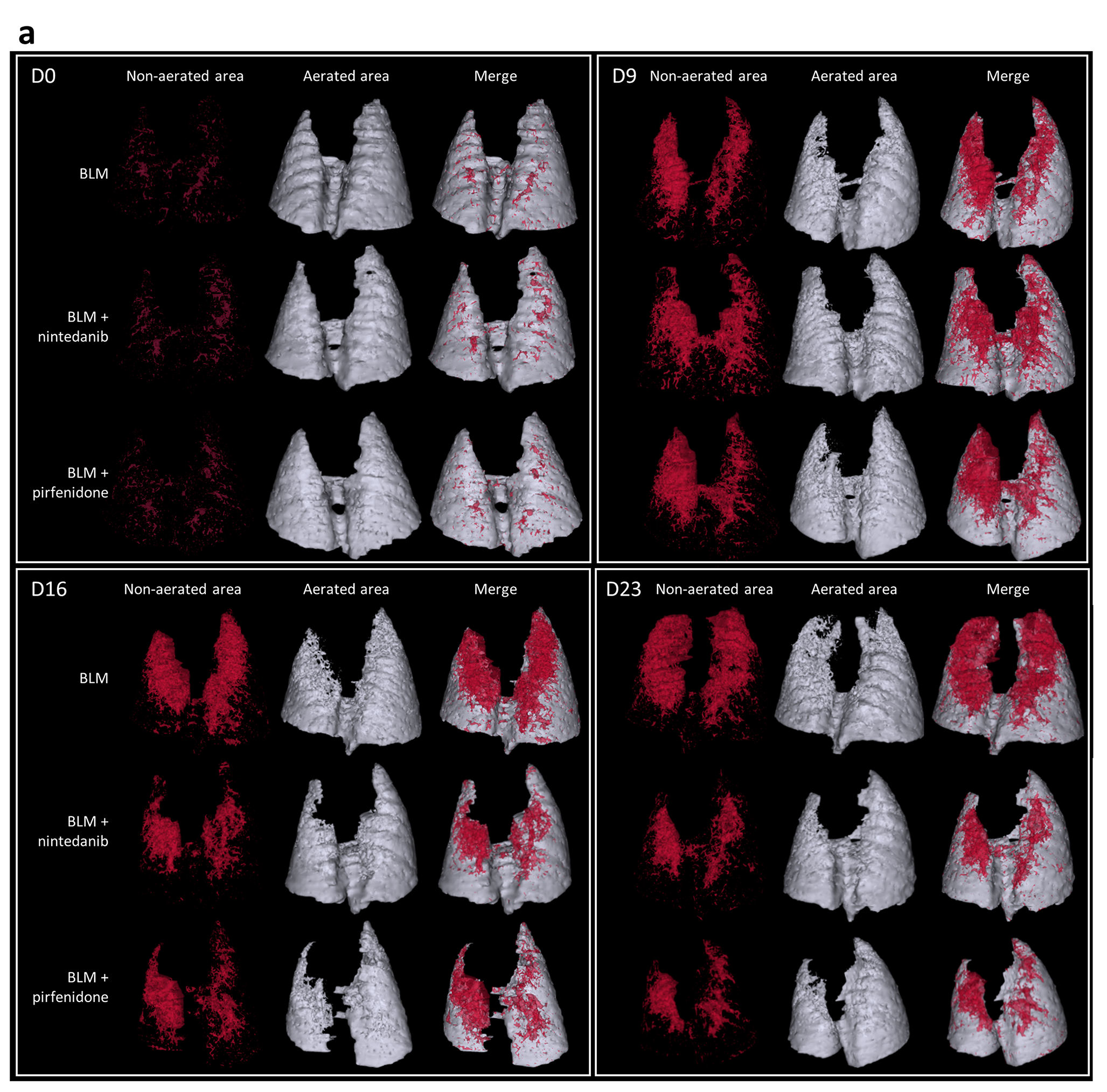

Supplement: Supplementary file 9 — a/ Representative 3D reconstruction of lung 3DROI segmentation of lung CT images of BLM-receiving mice treated or not with nintedanib of pirfenidone at D0, D9, D16 and D23. Red represents high density lung areas (-100 to 300 HU) representative of non-aerated lungs, gray represents normal density lung areas (-800 to -100 HU) representative of aerated lungs. (PNG 3272 kb) [file 259_2021_5209_Fig12_ESM.png]

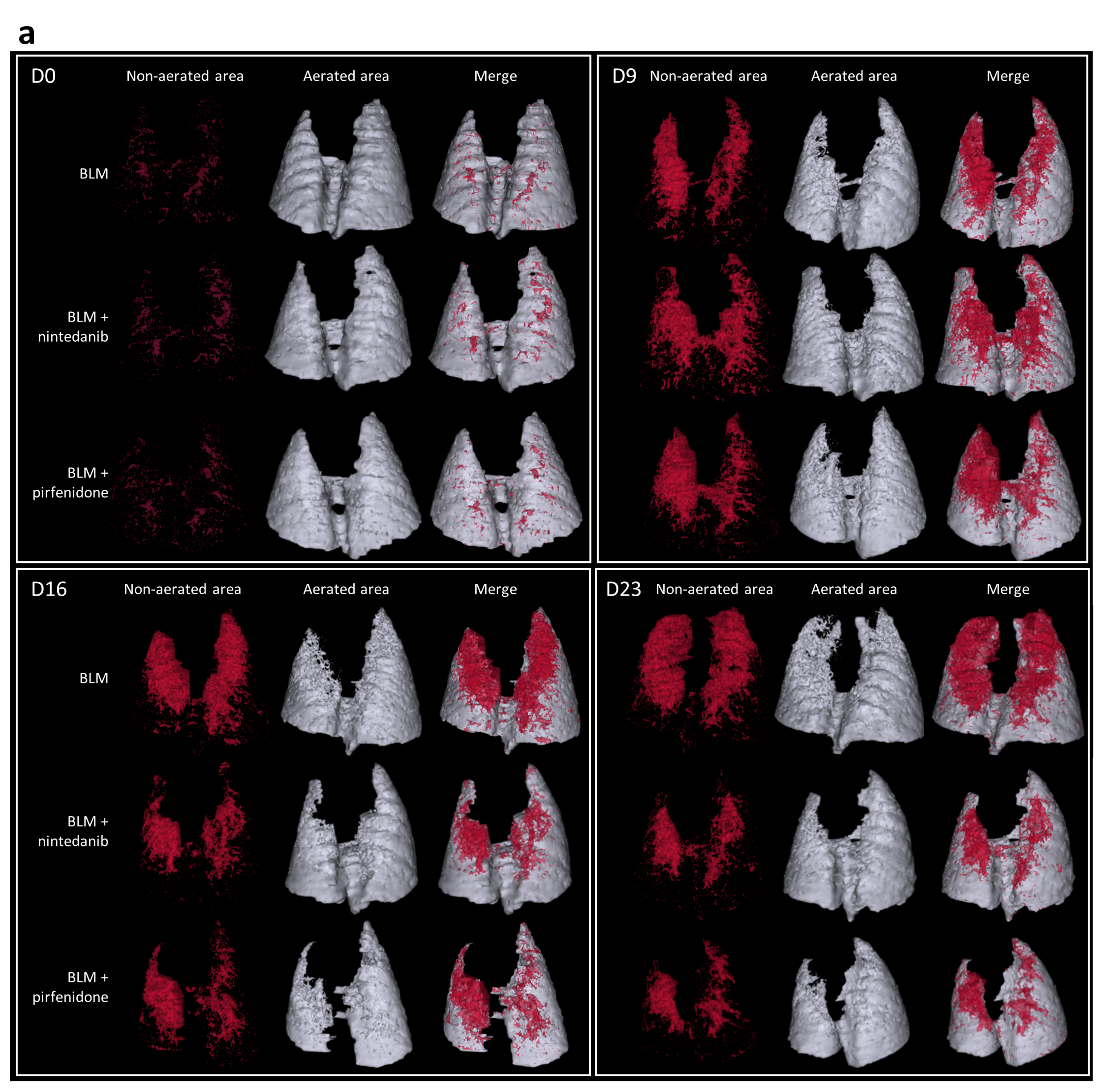

Supplement: Supplementary file 10 — High resolution image (TIF 11367 kb) [file 259_2021_5209_MOESM5_ESM.tif]

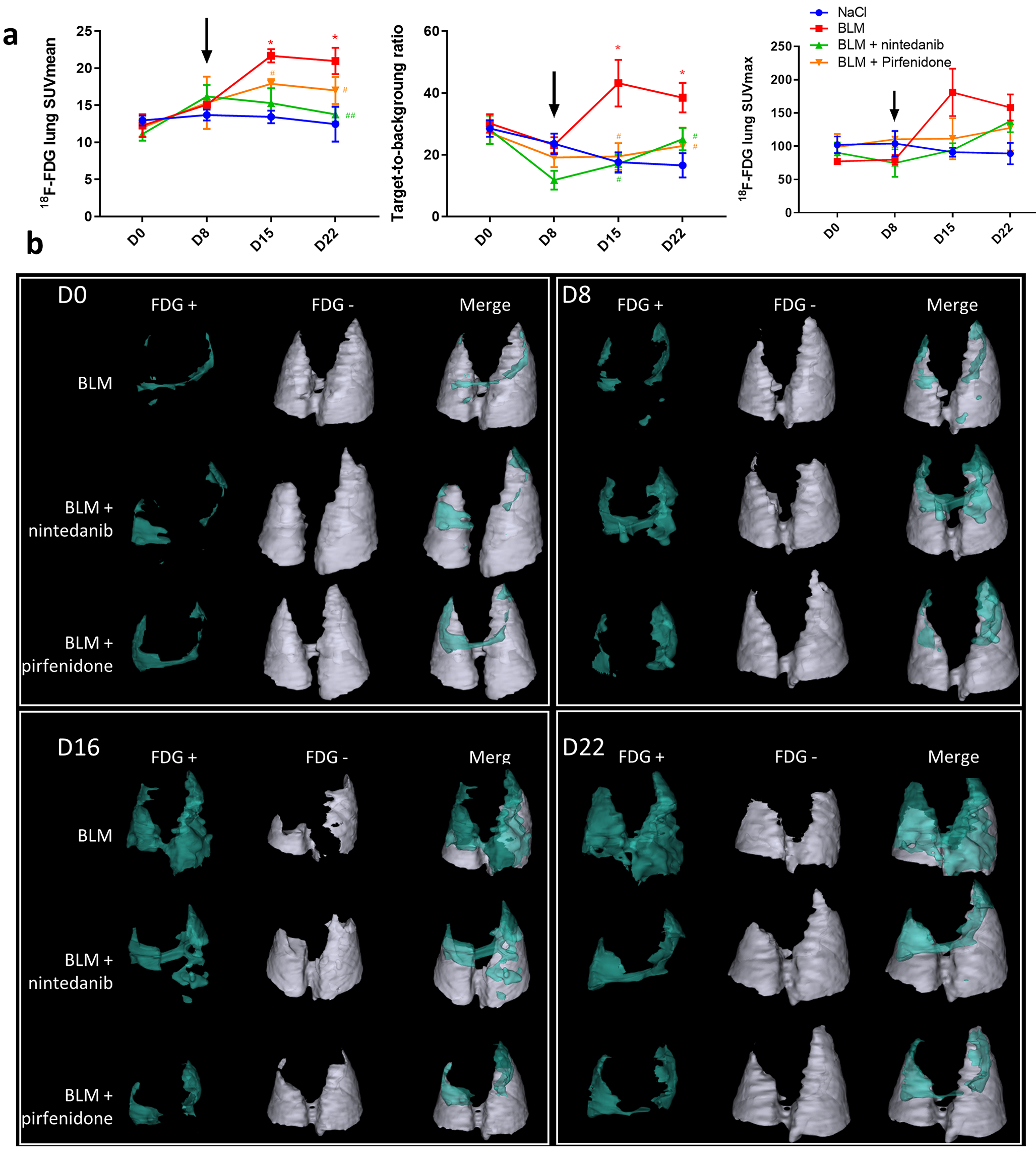

Supplement: Supplementary file 11 — a/ Graph represents evolution of 18F-FDG lung uptake in Nacl- and BLM- and receiving mice treated or not with nintedanib of pirfenidone at D0, D9, D15 and D22 in SUVmean (left), target to background ratio (center) and SUVmax (right). Results are presented as mean ± SEM, n = 4 for Nacl and n = 5 for other goups. Stars (*) are representative of statistical comparison between time points for each groups and hashs (#) are representative of statistical comparison between the groups at each time points. *(#)p<0.05, **(##)p<0.01. b/ Representative 3D reconstruction of lung 3DROI segmentation of 18F-FDG PET images representative of metabolic lung volume (MLV) of BLM-receiving mice treated or not with nintedanib of pirfenidone at D0, D8, D15 and D22. Cyan represents areas with high 18F-FDG lung uptake (above threshold of MLVthreshold = (SUVmean)D0 + 2SD), gray represents areas with low 18F-FDG lung uptake (below the threshold). (PNG 1786 kb) [file 259_2021_5209_Fig13_ESM.png]

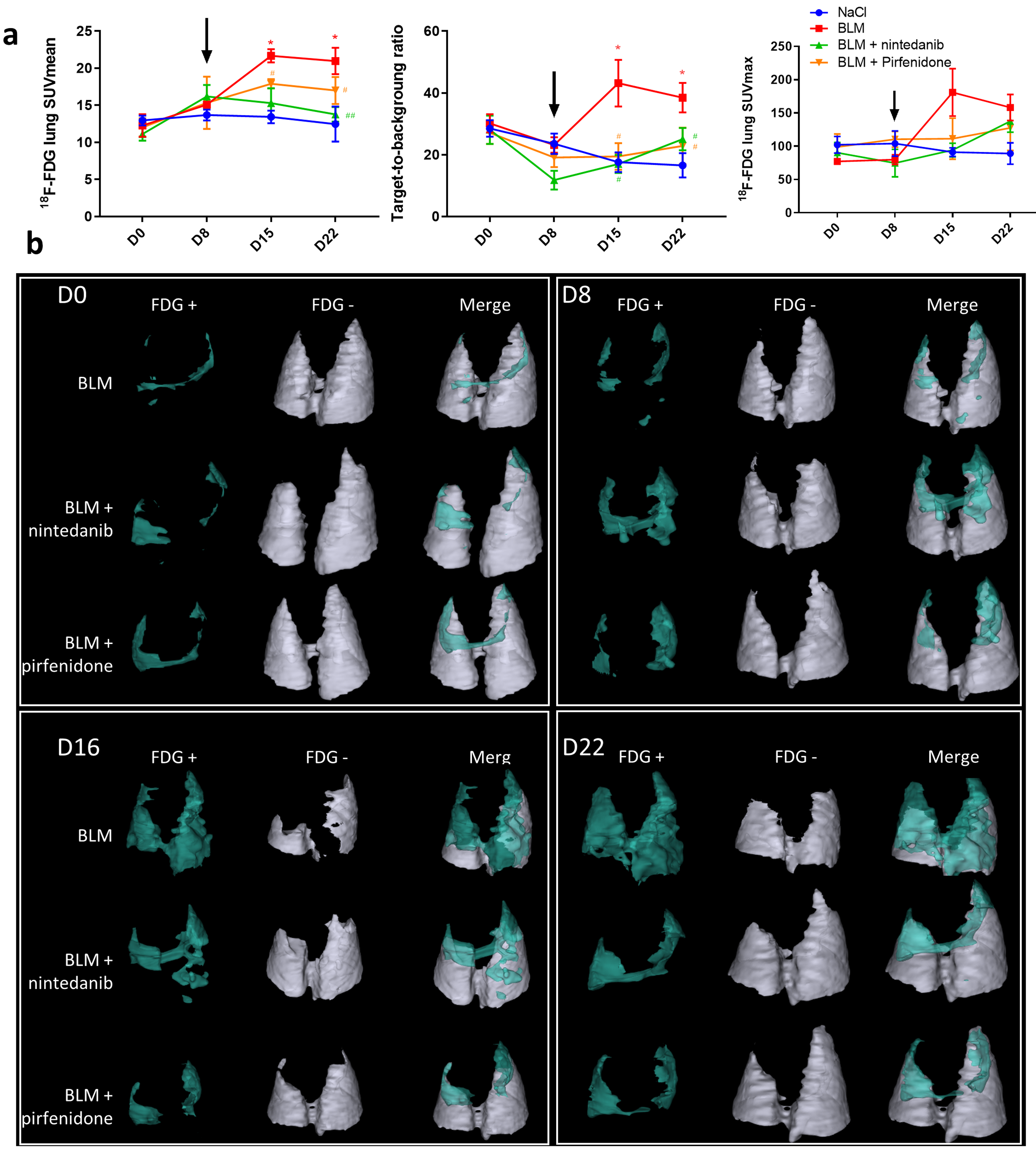

Supplement: Supplementary file 12 — High resolution image (TIF 5831 kb) [file 259_2021_5209_MOESM6_ESM.tif]

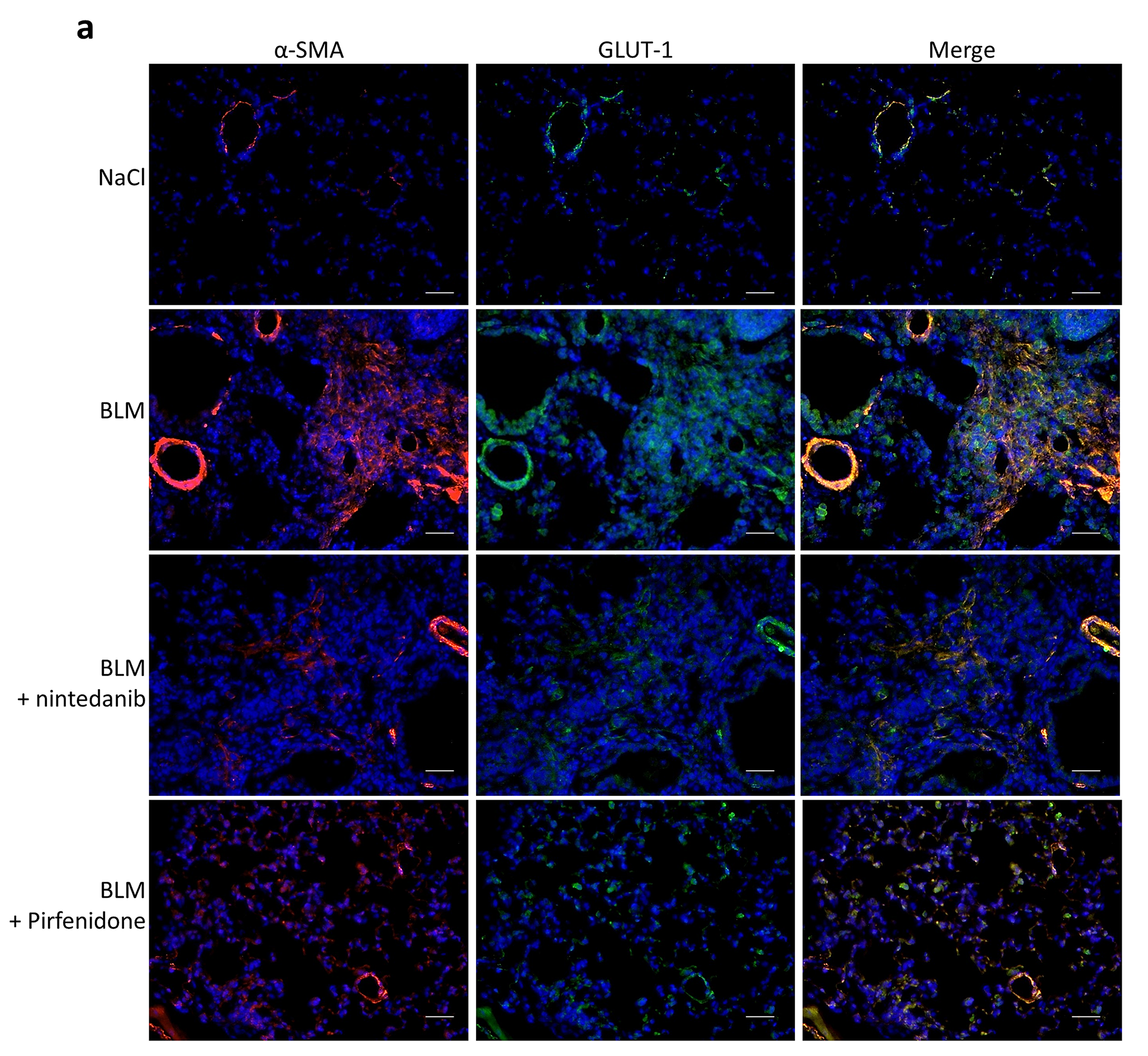

Supplement: Supplementary file 13 — a/ Immunofluorescence staining of α-SMA (red) and GLUT-1 (green) on lung section form Nacl- and BLM-receiving mice treated or not with nintedanib of pirfenidone at D21. Scale bars = 100 μm. (PNG 3979 kb) [file 259_2021_5209_Fig14_ESM.png]

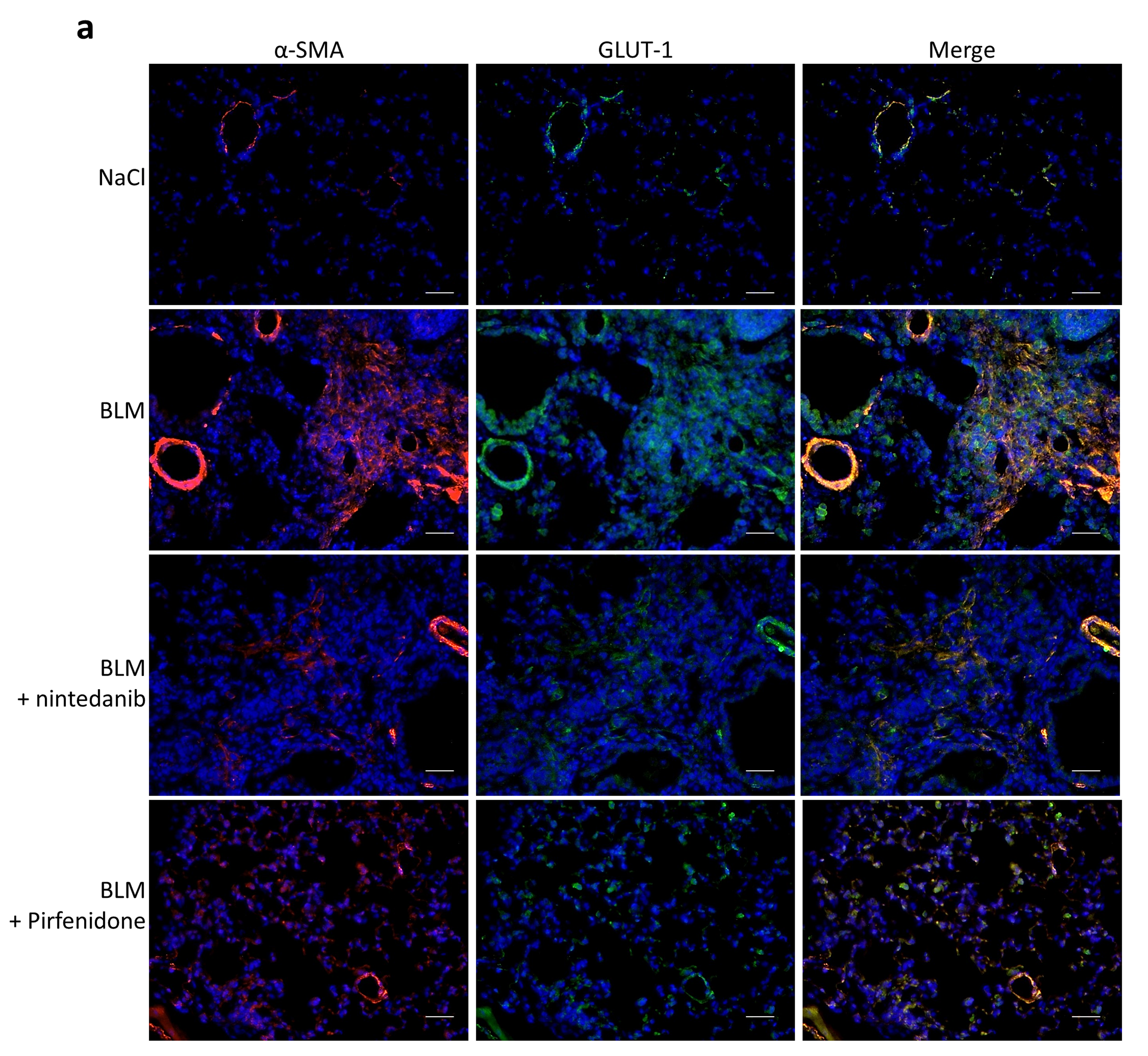

Supplement: Supplementary file 14 — High resolution image (TIF 14565 kb) [file 259_2021_5209_MOESM7_ESM.tif]

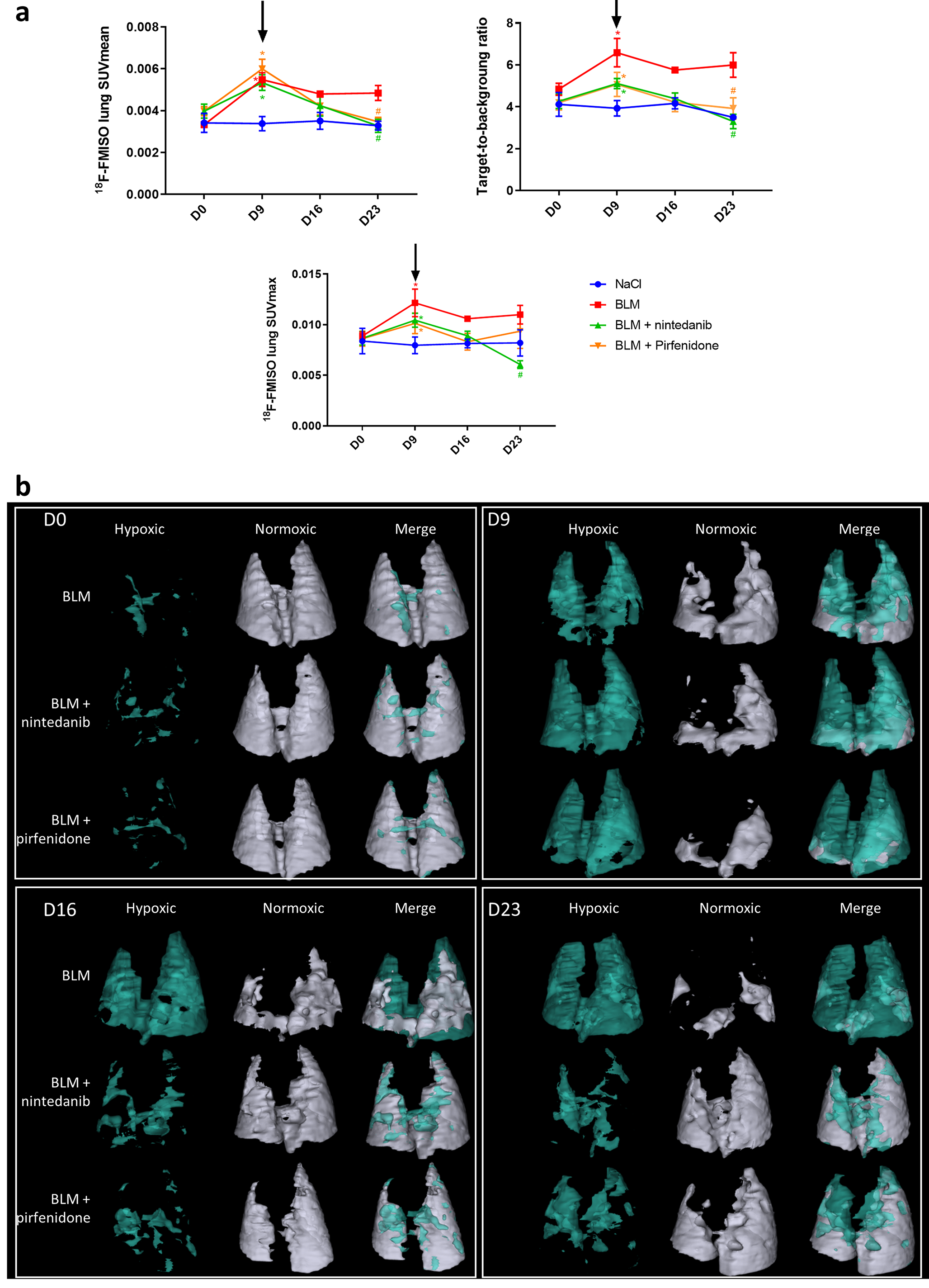

Supplement: Supplementary file 15 — a/ Graph represents evolution of 18F-FMISO lung uptake in Nacl- and BLM- and receiving mice treated or not with nintedanib of pirfenidone at D0, D9, D15 and D22 in SUVmean (left), target to background ratio (right) and SUVmax (lower). Results are presented as mean ± SEM, n = 4 for Nacl and n = 5 for other goups. Stars (*) are representative of statistical comparison between time points for each groups and hashs (#) are representative of statistical comparison between the groups at each time points. *(#)p<0.05. Black arrow represents the start of treatments. b/ Representative 3D reconstruction of lung 3DROI segmentation of 18F-FMISO PET images representative of hypoxic lung volume (HLV) of BLM-receiving mice treated or not with nintedanib of pirfenidone at D0, D9, D16 and D23. Cyan represents areas with high 18F-FMISO lung uptake (above threshold of HLVthreshold= TBR x 1.4), gray represents areas with low 18F-FMISO lung uptake (below the threshold). (PNG 2265 kb) [file 259_2021_5209_Fig15_ESM.png]

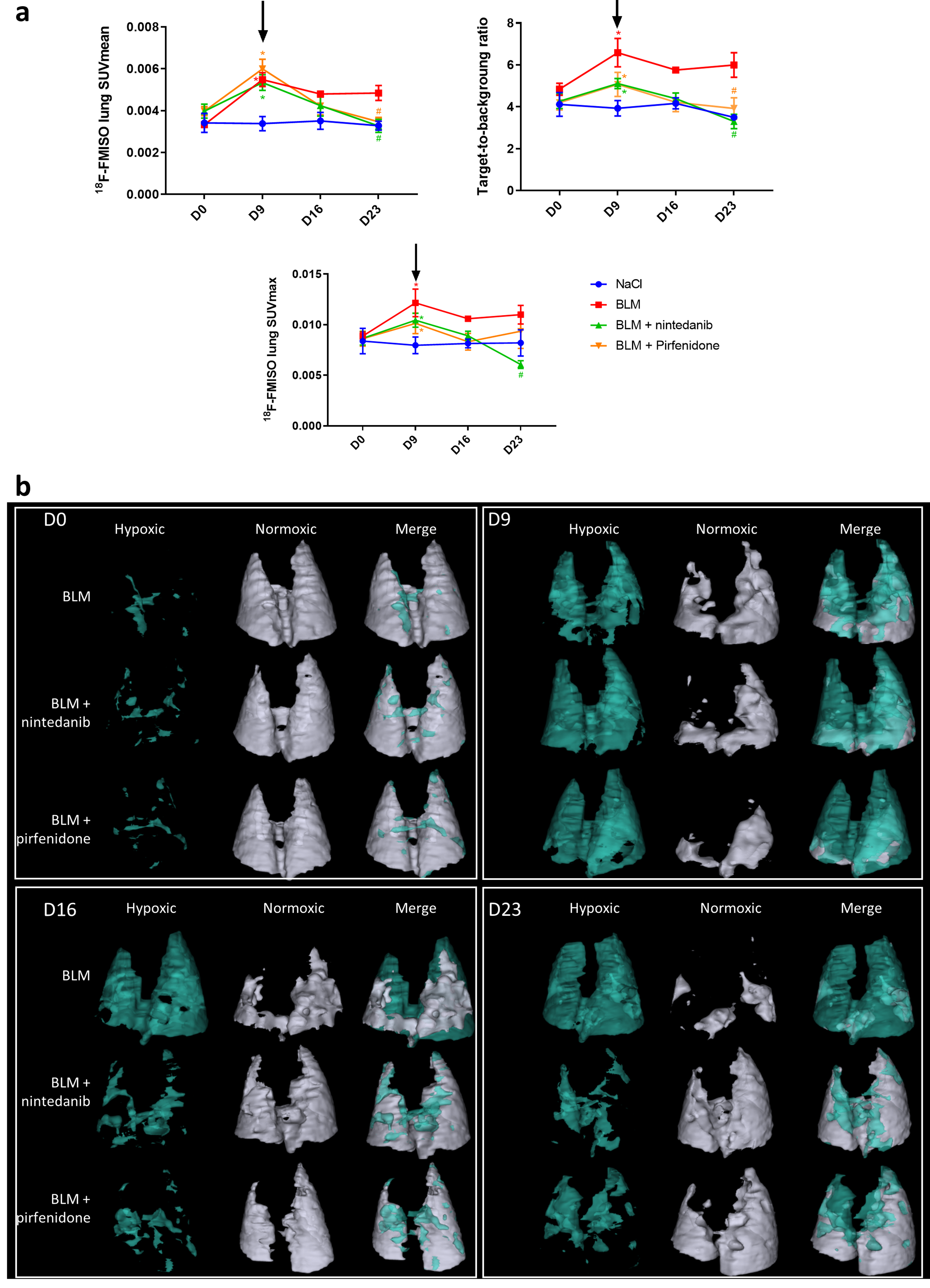

Supplement: Supplementary file 16 — High resolution image (TIF 7167 kb) [file 259_2021_5209_MOESM8_ESM.tif]

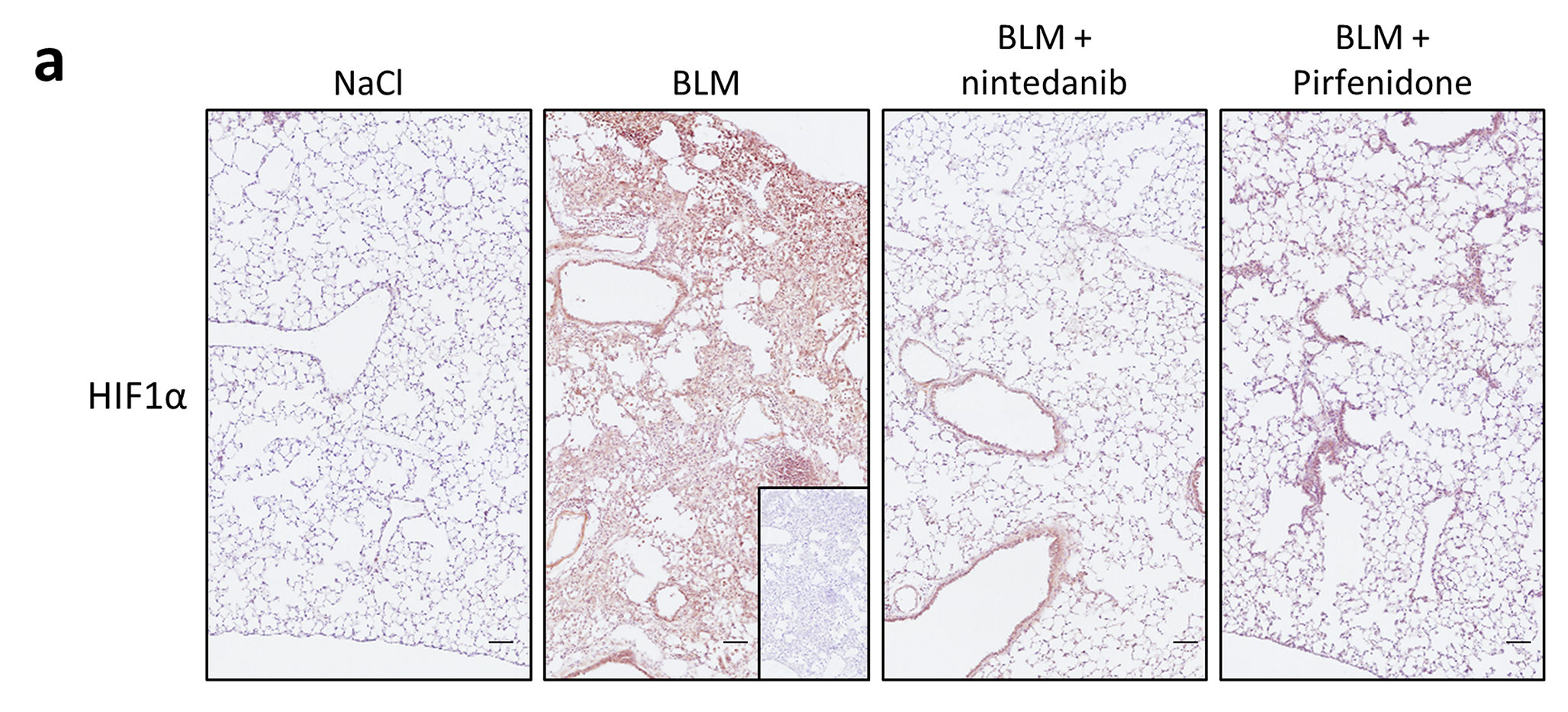

Supplement: Supplementary file 17 — a/ HIF-1α staining on lung section form Nacl- and BLM-receiving mice treated or not with nintedanib of pirfenidone at D21. Insert represent the isotype control. Scale bars = 100 μm. (PNG 2551 kb) [file 259_2021_5209_Fig16_ESM.png]

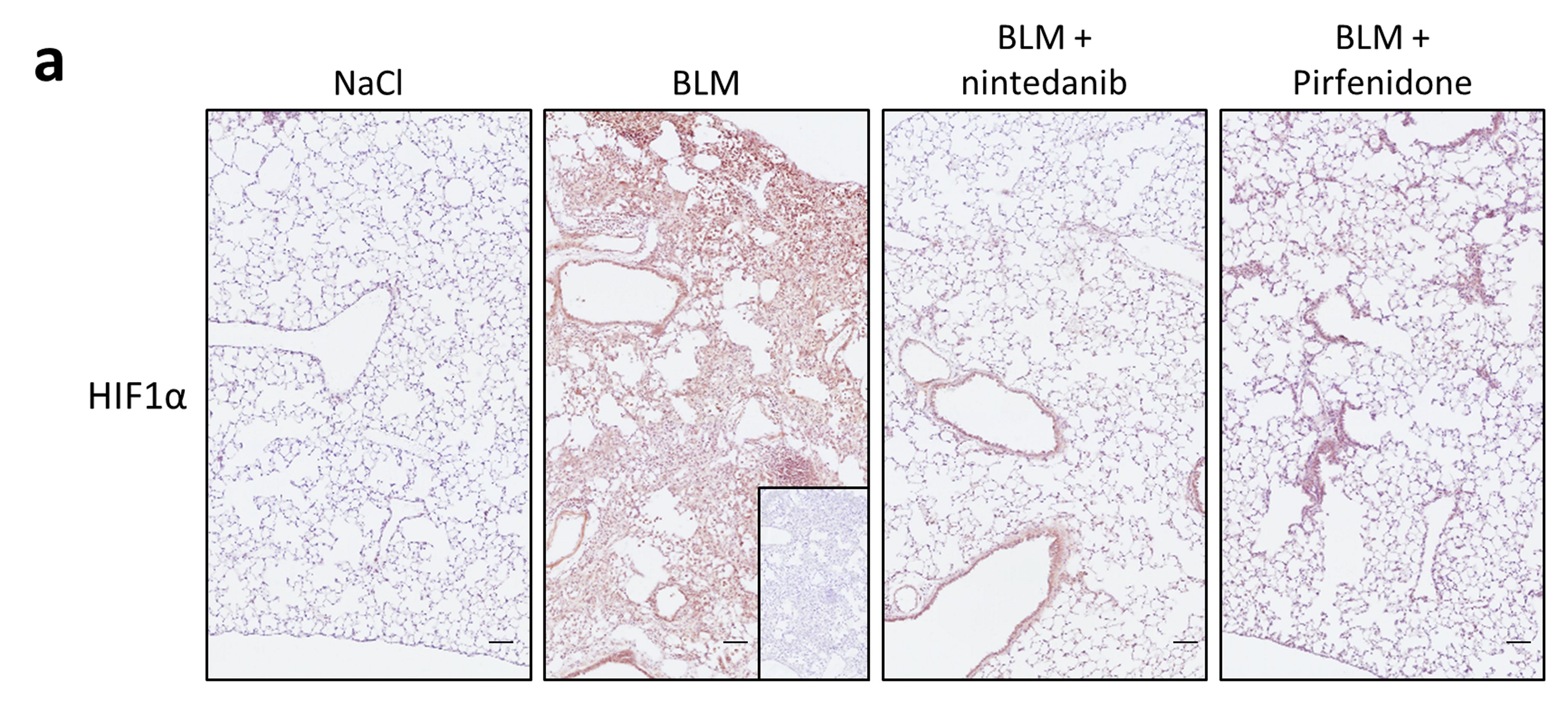

Supplement: Supplementary file 18 — High resolution image (TIF 8760 kb) [file 259_2021_5209_MOESM9_ESM.tif]

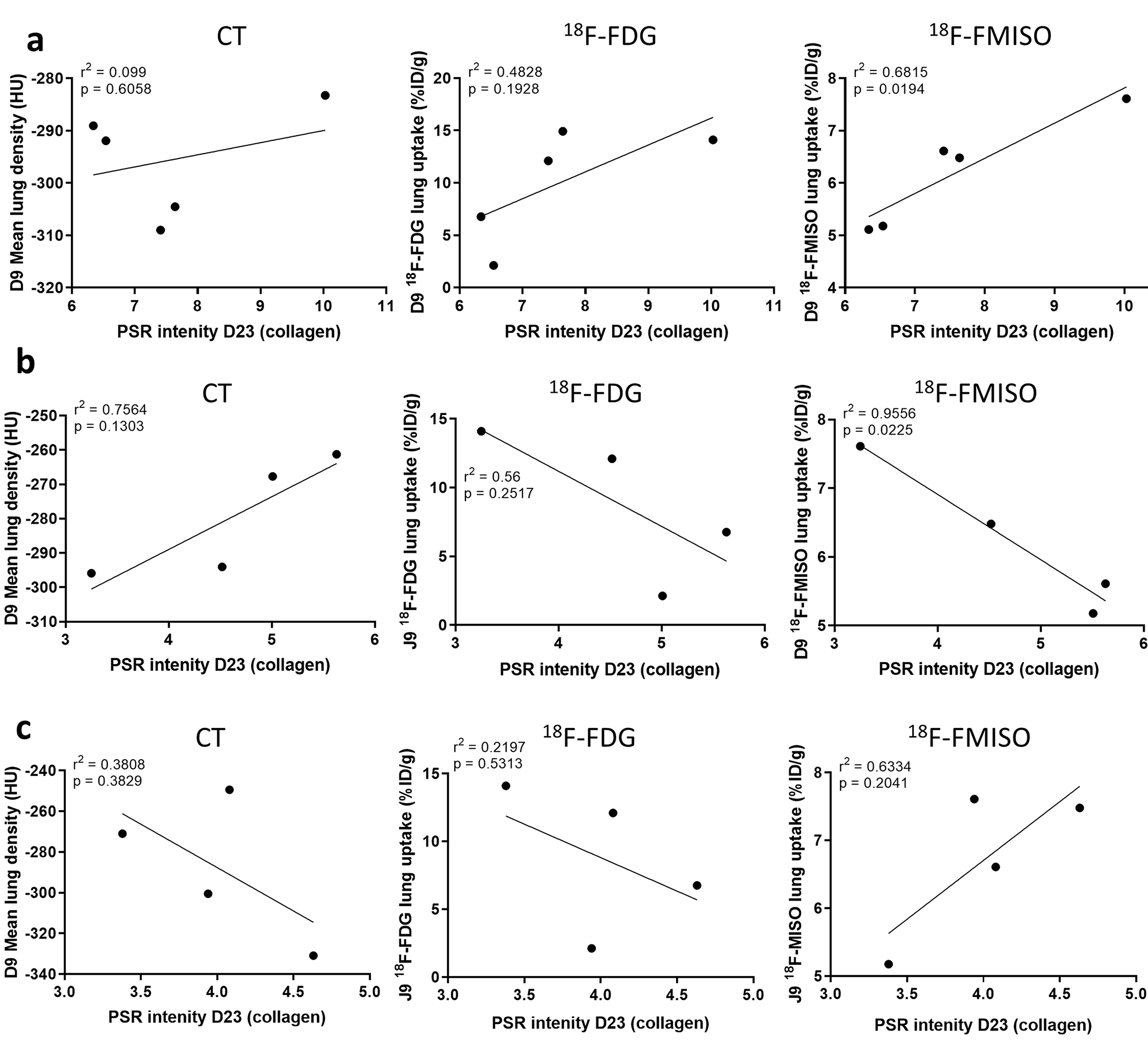

Supplement: Supplementary file 19 — a/ Correlation between, D9 mean lung density (left), D9 [18F]FDG lung uptake (center) or D9 [18F]FMISO lung uptake (right) and intensity of picrosirius red staining in BLM-receiving mice. b/ Correlation between, D9 mean lung density (left), D9 [18F]FDG lung uptake (center) or D9 [18F]FMISO lung uptake (right) and intensity of picrosirius red staining in BLM-receiving mice treated with nintedanib. c/ Correlation between, D9 mean lung density (left), D9 [18F]FDG lung uptake (center) or D9 [18F]FMISO lung uptake (right) and intensity of picrosirius red staining in BLM-receiving mice treated with pirfenidone. (PNG 525 kb) [file 259_2021_5209_Fig17_ESM.png]

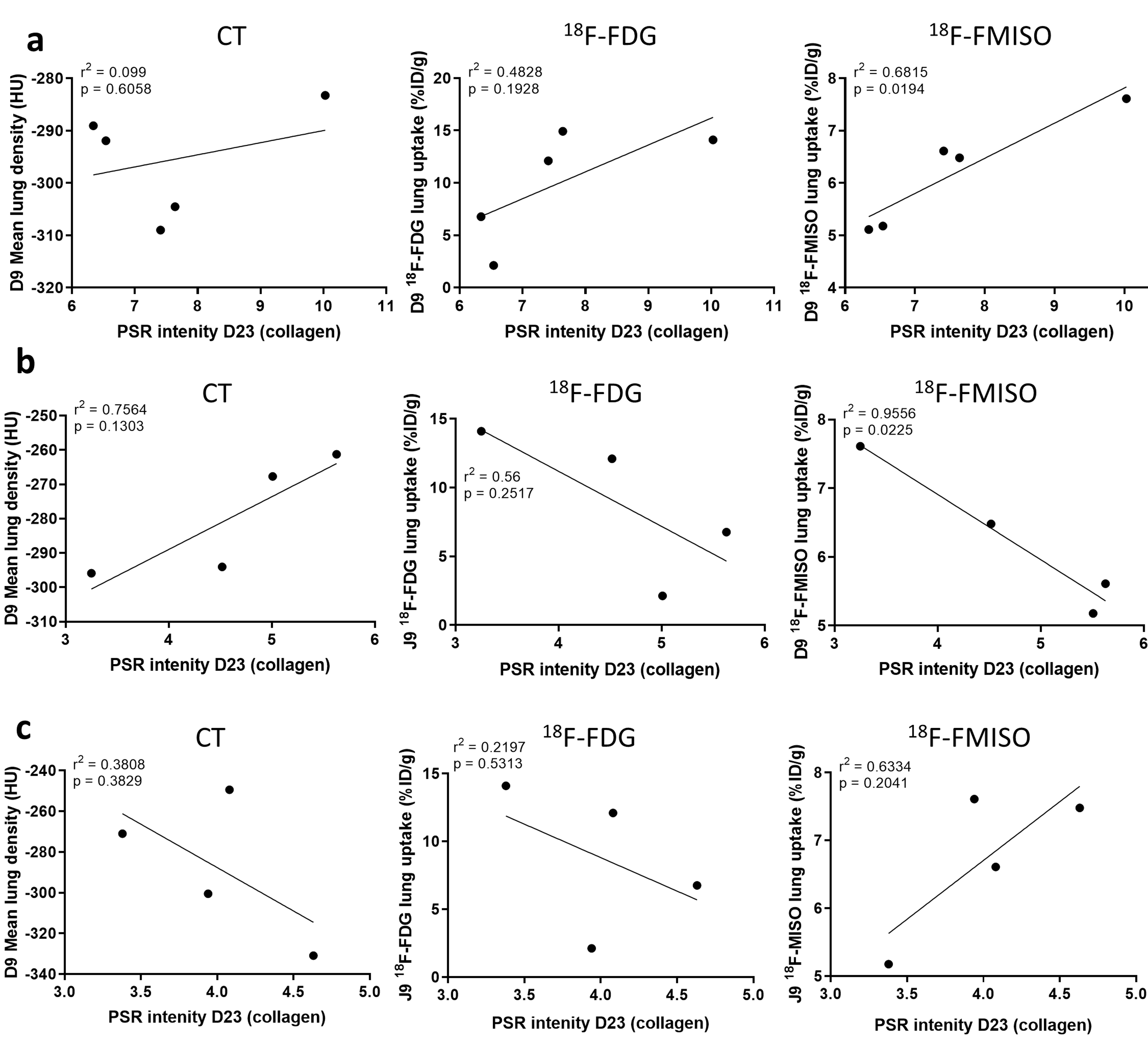

Supplement: Supplementary file 20 — High resolution image (TIF 1605 kb) [file 259_2021_5209_MOESM10_ESM.tif]

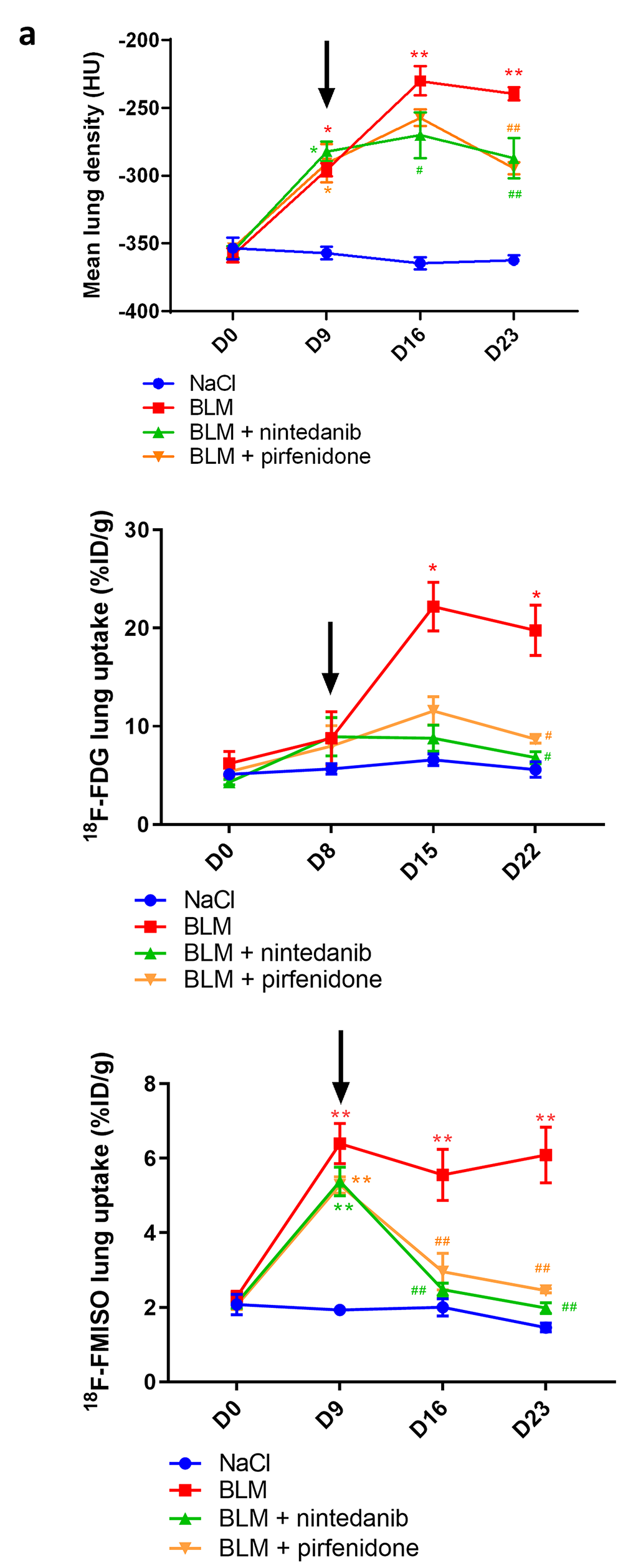

Supplement: Supplementary file 21 — a/ Time course of mean lung density, [18F]FMISO uptake and [18F]FDG uptake variations upon BLM and nintedanib/pirfenidone treatments. (PNG 318 kb) [file 259_2021_5209_Fig18_ESM.png]

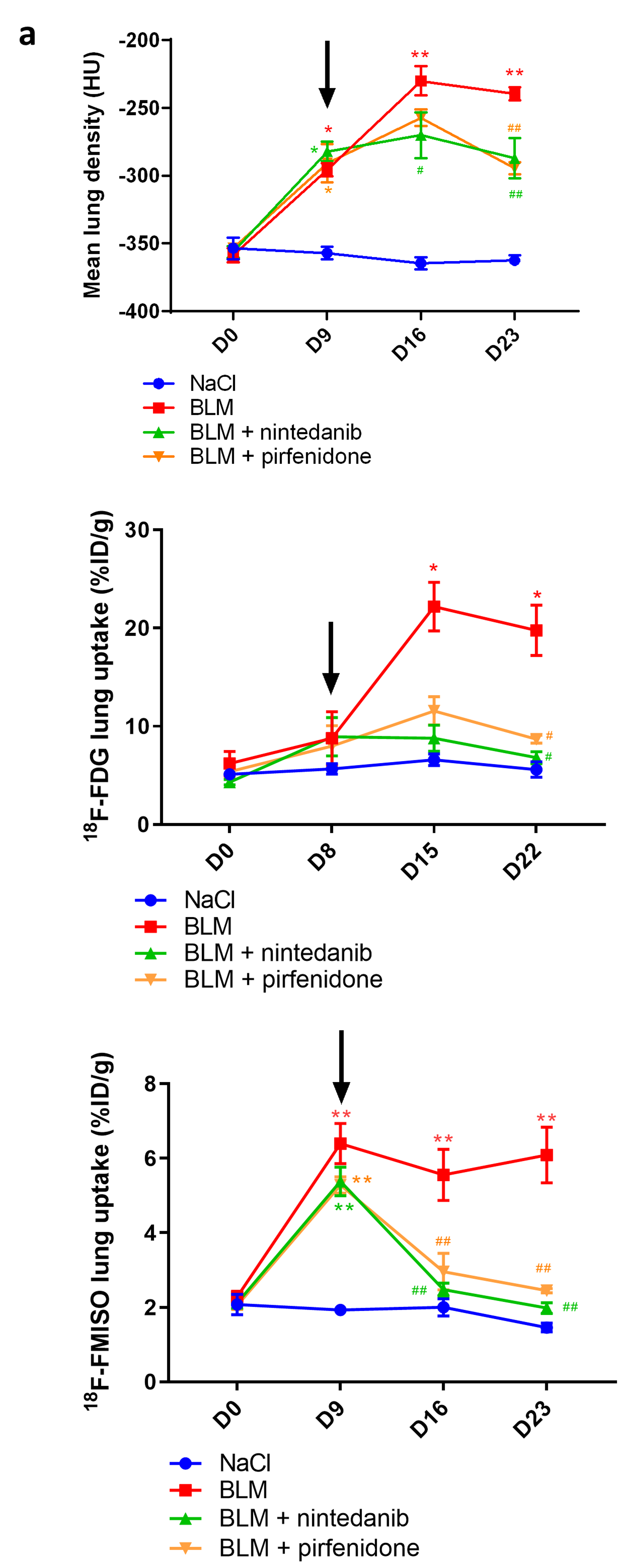

Supplement: Supplementary file 22 — High resolution image (TIF 951 kb) [file 259_2021_5209_MOESM11_ESM.tif]
